# COVID-19 diagnosis, vaccination during pregnancy, and adverse pregnancy outcomes of 865,654 women in England and Wales: a population-based cohort study

**DOI:** 10.1016/j.lanepe.2024.101037

**Published:** 2024-08-22

**Authors:** Elena Raffetti, Thomas Bolton, John Nolan, Luisa Zuccolo, Rachel Denholm, Gordon Smith, Ashley Akbari, Katie Harron, Gwenetta Curry, Elias Allara, Deborah A. Lawlor, Massimo Caputo, Hoda Abbasizanjani, Tim Chico, Angela M. Wood, CVD-COVID-UK/COVID-IMPACT Consortium and the Longitudinal Health and Wellbeing COVID-19 National Core Study

**Affiliations:** a British Heart Foundation Cardiovascular Epidemiology Unit, Department of Public Health and Primary Care, University of Cambridge, Cambridge, UK; b Victor Phillip Dahdaleh Heart and Lung Research Institute, University of Cambridge, Cambridge, UK; c Deparment of Global Public Health, Karolinska Institutet, Stockholm, Sweden; d Department of Earth Sciences, Uppsala University, Uppsala, Sweden; e British Heart Foundation Data Science Centre, Health Data Research UK, London, UK; f Health Data Science Centre, Human Technopole, Milan, Italy; g MRC Integrative Epidemiology Unit, University of Bristol, Bristol, UK; h Population Health Sciences, Bristol Medical School, University of Bristol, Bristol, UK; i NIHR Bristol Biomedical Research Centre, Bristol, UK; j Health Data Research UK South-West, Bristol, UK; k Department of Obstetrics and Gynaecology, University of Cambridge, Cambridge, UK; l Population Data Science, Swansea University Medical School, Faculty of Medicine, Health & Life Science, Swansea University, Swansea, UK; m Population, Policy & Practice Dept UCL GOS Institute of Child Health, London, UK; n Edinburgh Migration and Ethnicity Health Research Group, College of Medicine and Veterinary Medicine, Usher Institute, University of Edinburgh, UK; o Translational Health Science, Bristol Medical School, University of Bristol, Bristol, UK; p Division of Clinical Medicine, School of Medicine and Population Health, The Medical School, University of Sheffield, Beech Hill Road, Sheffield, UK; q National Institute for Health and Care Research Blood and Transplant Research Unit in Donor Health and Behaviour, University of Cambridge, Cambridge, UK; r Health Data Research UK Cambridge, Wellcome Genome Campus and University of Cambridge, Cambridge, UK; s Cambridge Centre of Artificial Intelligence in Medicine, UK

**Keywords:** Adverse pregnancy outcomes, COVID-19, Vaccination, Trimester, COVID-19 variants

## Abstract

**Background:**

The extent to which COVID-19 diagnosis and vaccination during pregnancy are associated with risks of common and rare adverse pregnancy outcomes remains uncertain. We compared the incidence of adverse pregnancy outcomes in women with and without COVID-19 diagnosis and vaccination during pregnancy.

**Methods:**

We studied population-scale linked electronic health records for women with singleton pregnancies in England and Wales from 1 August 2019 to 31 December 2021. This time period was divided at 8th December 2020 into pre-vaccination and vaccination roll-out eras. We calculated adjusted hazard ratios (HRs) for common and rare pregnancy outcomes according to the time since COVID-19 diagnosis and vaccination and by pregnancy trimester and COVID-19 variant.

**Findings:**

Amongst 865,654 pregnant women, we recorded 60,134 (7%) COVID-19 diagnoses and 182,120 (21%) adverse pregnancy outcomes. COVID-19 diagnosis was associated with a higher risk of gestational diabetes (adjusted HR 1.22, 95% CI 1.18–1.26), gestational hypertension (1.16, 1.10–1.22), pre-eclampsia (1.20, 1.12–1.28), preterm birth (1.63, 1.57–1.69, and 1.68, 1.61–1.75 for spontaneous preterm), very preterm birth (2.04, 1.86–2.23), small for gestational age (1.12, 1.07–1.18), thrombotic venous events (1.85, 1.56–2.20) and stillbirth (only within 14-days since COVID-19 diagnosis, 3.39, 2.23–5.15). HRs were more pronounced in the pre-vaccination era, within 14-days since COVID-19 diagnosis, when COVID-19 diagnosis occurred in the 3rd trimester, and in the original variant era. There was no evidence to suggest COVID-19 vaccination during pregnancy was associated with a higher risk of adverse pregnancy outcomes. Instead, dose 1 of COVID-19 vaccine was associated with lower risks of preterm birth (0.90, 0.86–0.95), very preterm birth (0.84, 0.76–0.94), small for gestational age (0.93, 0.88–0.99), and stillbirth (0.67, 0.49–0.92).

**Interpretation:**

Pregnant women with a COVID-19 diagnosis have higher risks of adverse pregnancy outcomes. These findings support recommendations towards high-priority vaccination against COVID-19 in pregnant women.

**Funding:**

BHF, ESRC, Forte, HDR-UK, MRC, NIHR and VR.

**Research in context**

**Evidence before this study**

We searched for prospective epidemiological studies of COVID-19 diagnosis and vaccination investigating adverse pregnancy outcomes published in any language up until Dec 1, 2023 (with no specified earliest date), in MEDLINE, Scientific Citation Index Expanded, and Embase using relevant terms ((‘pregnan’∗ OR ‘gestation’ OR ‘birth’) AND (‘diabetes’ OR ‘hypertens’∗ OR ‘preeclampsia’ OR ‘preterm’ OR ‘premature’∗ OR ‘pregnancy’ ‘complication’ OR (‘adverse’ AND ‘birth’ AND ‘outcome∗’) OR ‘stillbirth’ OR (‘pregnan’∗ AND ‘loss’) OR ‘cardiovascular’ OR (‘venous’ AND ‘thrombo∗’)) AND (‘COVID’ OR ‘SARS-CoV-2’ OR ‘coronavirus’)) (n = 9309). We found many primary reports and literature-based reviews showing COVID-19 diagnosis was associated with higher risks of common adverse pregnancy outcomes (e.g., gestational hypertensive disorders in pregnancy and preterm) and COVID-19 vaccination offered protection against adverse pregnancy outcomes. However, there was conflicting evidence about how COVID-19 diagnosis relates to more rare and serious outcomes including stillbirth and venous thrombosis. Few studies investigated how the risks are affected by the timing of the COVID-19 diagnosis and vaccination during pregnancy.

**Added value of this study**

Our study used population-scale linked electronic health records from 864,654 pregnant women in England and Wales to reliably examine the relationships between timing of COVID-19 diagnosis and vaccination and adverse pregnancy outcomes. This study afforded several advantages. First, it is the largest study to date, providing enhanced generalisabllity. Second, we studied a wide range of common and rare (including venous thrombosis and stillbirth) adverse outcomes after both COVID-19 infection and vaccination. Third, we conducted our analyses by pregnancy trimester, time since COVID-19 diagnosis and vaccination, before and after the vaccination rollout in England and Wales, and COVID-19 variant. Fourth, we adjusted for a wide-range of potential confounders available in electronic health records.

**Implications of all the available evidence**

The chief implication of this study for public policy is to support recommendations towards high-priority vaccination against COVID-19 in pregnant women to avoid high risks of adverse pregnancy outcomes from COVID-19 especially in the 3rd trimester. The chief implication for scientific understanding is the novel evidence about the associations between timing of COVID-19 and common and rare adverse pregnancy outcomes, which are stronger within 14-days since COVID-19 infection, when COVID-19 occurs in the 3rd trimester and which remain even in the vaccination roll-out era.

## Introduction

The physiological maternal adaptations to pregnancy, such as expansion of blood volume, increase of insulin resistance, and immunological changes, can unmask a latent predisposition in a woman to cardiovascular and cardiometabolic complications, such as pre-eclampsia, gestational hypertension, and gestational diabetes. These complications can, in turn, affect fetal development, leading to growth restriction (as for pre-eclampsia), overgrowth (associated with gestational diabetes), and preterm birth, often requiring medical intervention and increasing the risk of stillbirth.^1^ Additional viremia and inflammatory phases of respiratory virus-related infections such as H1N1 influenza, and more recently COVID-19 may further hamper maternal adaptation inducing pro-coagulation state, affecting uteroplacental circulation and, consequently, fetal growth.^2^, ^3^, ^4^, ^5^, ^6^, ^7^, ^8^, ^9^, ^10^, ^11^, ^12^, ^13^, ^14^

Studies investigating COVID-19 in pregnant women have largely focused either on COVID-19 infected women alone or on comparisons between COVID-19 infected and non-infected women at the time of birth.^10^^,^^15^, ^16^, ^17^, ^18^, ^19^, ^20^, ^21^, ^22^, ^23^, ^24^ Few population-wide studies and multi-cohort studies, including a study from Denmark (n = 111,186)^13^ and Canada (n = 6012),^7^ and a multi-national cohort study involving 18 countries,^9^ examined COVID-19 during pregnancy. These studies showed that pregnant women diagnosed with COVID-19 are more likely to experience pre-eclampsia,^8^^,^^12^^,^^13^^,^^25^ gestational hypertension^9^ and preterm birth.^7^^,^^8^^,^^12^, ^13^, ^14^^,^^26^ Whilst a meta-analysis of 117,552 vaccinated pregnant women underscores COVID-19 vaccine efficacy in preventing infection and hospitalization,^27^^,^^28^ few studies have addressed the safety and effectiveness of COVID-19 vaccination during pregnancy.^22^^,^^23^^,^^28^, ^29^, ^30^, ^31^, ^32^ Current studies also overlook, with few exceptions,^8^^,^^12^^,^^13^^,^^33^ the relationship between COVID-19 diagnosis and vaccination during pregnancy, including the risks of rare outcomes (such as stillbirth and venous thrombotic events), and how risks differ by time since infection or vaccination, pregnancy trimester or secular time period (e.g., accounting for changes in COVID-19 variants and vaccination rollouts).

To address these gaps, we leveraged population-wide linked electronic health record (EHR) data sources from England and Wales to build the largest population-wide pregnancy cohort of women with a record of birth during the COVID-19 pandemic period. In our study we compared the incidence of adverse pregnancy outcomes in women with and without COVID-19 diagnosis and vaccination during pregnancy stages, accounting for a wide range of potential confounding factors. Our aim was to provide quantitative evidence for recommendations concerning antenatal care of women during their pregnancy and their vaccination against current and future COVID-19 outbreaks.

## Methods

### Study setting and population

A population-wide pregnancy cohort was defined using Hospital Episode Statistics (HES) maternity information in England and the Maternity Indicator Dataset (MIDS) in Wales. We included 865,654 women with (i) a singleton birth episode, (ii) estimated pregnancy start date for the 1st record of birth after 1st August 2019 and with a delivery date before 31st December 2021, and (iii) registered with a primary care general practice in England or Wales at the estimated pregnancy start date. The pregnancy cohort was linked with primary care events, emergency events, hospital admissions, critical care admissions, outpatient visits, COVID-19 test results, community dispensing records, and deaths. For women without a recorded estimated gestational age at delivery (n = 200,020, 23.1%), we considered a standard pregnancy duration of 280 days. See Supplementary Methods for more detail in the definition of the pregnancy cohort.

The pregnancy cohort was divided at 8th December 2020 (the start of the vaccination programme in the UK) into two subcohorts of pregnancies during pre-vaccination and vaccination roll-out eras. The subcohort for the pre-vaccination era included women with an estimated pregnancy start date from 1st August 2019, and delivery date up to 8th December 2020. The subcohort for the vaccination era included women with an estimated pregnancy start date after 8th December 2020 and delivery date up to 31st December 2021. Pregnancies that spanned across both periods were excluded from the subcohorts.

We categorized the periods of the COVID-19 pandemic into original, alpha, and delta eras, based on the prevalent strain: original variant: 1st July 2020 to 7th December 2020; alpha variant: 8th December 2020 to 17th May 2021; delta variant: 18th May 2021 to 13th December 2021.^34^

Data were accessed in National Health Service (NHS) England's Secure Data Environment (SDE) service for England^35^ and in the Secure Anonymised Information Linkage (SAIL) Databank for Wales, via the British Heart Foundation Data Science Centre's CVD-COVID-UK/COVID-IMPACT Consortium. The analysis was performed according to a prespecified protocol. The phenotypes and associated code is available at https://github.com/BHFDSC/CCU018_01. The study is reported in agreement with the RECORD and STROBE statements for observational studies using routinely collected health data.

### COVID-19 diagnosis

COVID-19 diagnosis was defined at the earliest recorded date of a positive COVID-19 polymerase chain reaction (PCR) or antigen test or a confirmed COVID-19 diagnosis found in either primary care event records or secondary care hospital admission records, as defined in previous analyses.^36^ Hospitalisation related to COVID-19 was defined as a hospital admission record with a COVID-19 diagnosis code in the primary position within 4 weeks of from COVID-19 diagnosis as defined above.

### COVID-19 vaccination

COVID-19 vaccination during pregnancy was defined as having received dose 1 or dose 1 and dose 2 of ChAdOx1-S vaccine, BNT162b2 vaccine or a mix of these vaccines during pregnancy.

### Adverse pregnancy outcomes

We examined the following adverse pregnancy outcomes: gestational diabetes, gestational hypertension, pre-eclampsia, preterm birth (<37 weeks of pregnancy), very preterm birth (<32 weeks of pregnancy), small-for-gestational-age (<5th percentile), stillbirth and venous thrombotic events. The definition of each condition was based on rule-based phenotyping algorithms using SNOMED-CT (Systematised Nomenclature of Medicine–Clinical Terms), Read V2 (Read Coded Clinical Terms) and ICD-10 (International Classification of Diseases 10th Revision) (more details on the description of outcomes and data-sources in Table S1 and https://github.com/BHFDSC/CCU018_01).

### Confounders

We pre-specified the following as confounders based on them being known or plausibly associated with COVID-19 diagnosis/vaccination and adverse pregnancy outcomes: age (years), deprivation at residential area level (quintiles), calendar week, region (England only), ethnic group (categories of White, other ethnic groups, unknown ethnic group), previous pregnancy (yes/no), smoking status (categories of smoker, former smoker, non-smoker), history of hypertension (yes/no), history of diabetes (yes/no), history of haematological and cardiovascular diseases (yes/no), overweight/obesity (yes/no), history of depression (yes/no) and other conditions (yes/no, including at least one of chronic obstructive pulmonary disease, liver disease, chronic kidney disease, cancer and surgical intervention). All confounders were defined using primary and secondary care records and occured before the estimated pregnancy start date. Individuals with missing values for deprivation levels (0.4%) and smoking status (6.1%) were excluded from the analysis.

### Statistical analysis

We estimated nation-specific hazard ratios (HRs) in England and Wales, comparing the incidence of adverse pregnancy outcomes after a diagnosis of COVID-19 during pregnancy with the incidence of these outcomes in women before or without a diagnosis of COVID-19 during pregnancy. We estimated HRs in separate time periods (in days: [0,14), [14+,]) after diagnosis of COVID-19 and, separately, by pregnancy trimester of diagnosis (1st trimester ≤84 estimated pregnancy duration in days, 2nd trimester >84 < days ≤182, 3rd trimester >182) and by COVID-19 variants (original, alpha and delta era). Analyses used Cox regression models with estimated gestational age in days as the time scale and the estimated pregnancy start date as the time origin, fitted separately by nation. Censoring was at the earliest of the date of the outcome, delivery date or end of cohort follow-up date (i.e., 8th December 2020 or 31st December 2021). Nation-specific HRs for England and Wales were combined using inverse-variance weighted meta-analyses with fixed effect models. We visually assessed the proportional hazards assumption within each of these time periods for the main outcomes using log–log plots (see Supplementary Methods for more detail); there was no evidence of strong violation. To quantify the effectiveness of dose 1 of COVID-19 vaccine during pregnancy, we estimated nation-specific HRs, comparing the incidence of COVID-19 diagnosis in pregnant women after dose 1 of COVID-19 vaccine during pregnancy with the incidence of COVID-19 diagnosis in pregnant women before or without dose 1 of COVID-19 vaccine during pregnancy. Similarly, amongst pregnant women who had received dose 1 of COVID-19 vaccine during pregnancy, we estimated nation-specific HRs, comparing the incidence of COVID-19 diagnosis in women after dose 2 of COVID-19 vaccine during pregnancy with the incidence of COVID-19 diagnosis in women before or without dose 2 of COVID-19 vaccine. To quantify the association of dose 1 of COVID-19 vaccine during pregnancy and adverse pregnancy outcomes we estimated nation-specific HRs, comparing the incidence of adverse pregnancy outcomes after dose 1 of COVID-19 vaccine during pregnancy with the incidence of these outcomes in pregnant women before or without dose 1 of COVID-19 vaccine during pregnancy. Similarly, amongst pregnant women who had received dose 1 of COVID-19 vaccine during pregnancy, we estimated HRs comparing the incidence of adverse pregnancy outcomes after dose 2 of COVID-19 vaccine during pregnancy with the incidence of these outcomes in pregnant women before or without dose 2 of COVID-19 vaccine. Analyses used Cox regression models as described above, and were restricted to women with a pregnancy start date from 9th December 2020 and a delivery date up to 31st December 2021 and without a COVID-19 vaccination before pregnancy. In analyses of dose 2 of COVID-19 vaccine, the time origin was defined as the date of receiving the dose 1 of COVID-19 vaccine.

HRs were adjusted for (i) age and deprivation and (ii) age, deprivation, calendar week, region (England only) and a propensity score (spline term with 3 knots, with knots placed at the 25th, 50th, and 75th percentiles) incorporating all other available confounders. In analyses of dose 2 of COVID-19 vaccine, we further adjusted for the time between the estimated pregnancy start date and date of receiving dose 1 of COVID-19 vaccine. The propensity scores were estimated using logistic regression models; further details are provided in the Supplementary Methods.

We conducted subgroup analyses by COVID-19 vaccination status, (receiving two or more vaccine doses vs receiving fewer than 2 vaccine doses) age group (18–29/30–39/40–55 years), ethnic group (six categories: Black or Black British, Asian or Asian British, White, Mixed, Ethnic minorities and Unknown ethnic group), deprivation index, previous pregnancy, history of COVID-19 diagnosis before pregnancy and existing health conditions (i.e., at least one condition of haematological diseases, cardiovascular disease, hypertensive disorders, diabetes disorders, chronic obstructive pulmonary disease, liver disease, chronic kidney disease, cancer and surgical intervention during the last year before pregnancy). We also conducted a series of sensitivity analyses as specified in detail in Table S2. These included analyses restricted to women with known gestation age; with an estimated pregnancy start date <11th March 2020; with an estimated pregnancy start date ≥11th March 2020; who had not received any doses of COVID-19 vaccine up to 31st December 2021; who had received at least one dose of COVID-19 vaccine up to 31st December 2021 and who had received at least one dose of COVID-19 vaccine from 18th June 2021 to 31st December 2021 and in analyses which included outcomes occurring from day 1 (instead of day 0) of follow-up, when follow-up was censored at the maximal outcome week for preterm birth and very preterm birth and for spontaneous preterm births (rather than planned and spontaneous).

Results from analyses involving subgroups with less than ten incident cases are not reported, adhering to policies and processes of the NHS England SDE and the SAIL Databank.

Analyses used SQL, Python, and RStudio Version 1.3.1093.1 driven by R Version 4.0.3. Codelists used to define phenotypes (for eligibility, outcome and confounder information) are available at https://github.com/BHFDSC/CCU018_01.

The North East–Newcastle and North Tyneside 2 research ethics committee provided ethical approval for the CVD-COVID-UK research program (REC no. 20/NE/0161) to access, within secure trusted research environments, unconsented, whole-population, de-identified data from EHRs collected as part of patients’ routine healthcare.

### Role of the funding source

The funder of the study had no role in study design, data collection, data analysis, data interpretation, or writing of the report.

## Results

### COVID-19 diagnosis analysis

Of the 865,654 pregnant women eligible for analysis (829,180 in England and 36,474 in Wales, Figure S1), 60,134 (6.9%) women had a diagnosis of COVID-19 during pregnancy (Table 1). Median age was 30 years and 551,787 (63.7%) had a previous pregnancy, 84,740 (9.8%) had a history of hypertension, 9590 (1.1%) had a history of cardiovascular or haematological diseases and 44,549 (5.1%) had a history of diabetes. Characteristics of pregnant women were similar in the pre-vaccination and vaccination eras, and in those with and without COVID-19 diagnosis during pregnancy with few exceptions (Table 1 and Table S3): in the vaccination era there was a higher proportion of COVID-19 diagnosis than in the pre-vaccination era. Characteristics were comparable between Wales and England, with a few exceptions (Table S4): pregnant women were generally younger, more likely to be current smokers, have a history of depression and less ethnically diverse in Wales than England.

**Table 1:** Characteristics of the pregnancy cohort in the overall period, pre-vaccination era and vaccination era in England and Wales.

Characteristic	Overall, N = 865,654 n (%)	Pre-vaccination era, N = 318,192 n (%)	Vaccination era, N = 162,733 n (%)
**Age (years)**			
**Median (interquartile range)**	30 (26–34)	30 (26–33)	30 (26–34)
<30	403,318 (46.6)	152,268 (47.9)	73,590 (45.2)
30–39	434,849 (50.2)	156,033 (49)	83,577 (51.4)
≥40	27,487 (3.2)	9891 (3.1)	5571 (3.4)
**Deprivation**			
1 (least)	214,763 (24.8)	79,462 (25.5)	39,026 (24)
2	190,090 (22)	69,368 (22.3)	35,473 (21.8)
3	167,387 (19.3)	60,019 (19.3)	31,411 (19.3)
4	152,663 (17.6)	53,482 (17.2)	29,464 (18.1)
5 (most)	136,868 (15.8)	47,612 (15.3)	26,557 (16.3)
Unknown	3883 (0.4)	1428 (0.5)	807 (0.5)
**Ethnic group**			
Black or Black British	40,784 (4.7)	15,018 (4.7)	7745 (4.8)
Asian or Asian British	105,156 (12.1)	38,979 (12.3)	19,624 (12.1)
White	647,493 (74.8)	236,290 (74.3)	122,260 (75.1)
Mixed	19,219 (2.2)	6885 (2.2)	3752 (2.3)
Ethnic minorities	39,968 (4.6)	15,593 (4.9)	6953 (4.3)
Unknown ethnic group	13,029 (1.5)	5427 (1.7)	2399 (1.5)
**Previous pregnancy**	551,787 (63.7)	201,725 (63.4)	102,611 (63.1)
**COVID-19 diagnosis during pregnancy**	60,134 (6.9)	6821 (2.1)	17,260 (10.6)
**COVID-19 Hospitalization**	3626 (0.4)	451 (0.1)	1292 (0.8)
**Trimester of exposure**			
1st	11,250 (1.3)	175 (0.1)	3197 (2)
2nd	18,532 (2.1)	673 (0.2)	5558 (3.4)
3rd	30,352 (3.5)	5973 (1.9)	8505 (5.2)
**Dose 1 of COVID-19 vaccine during pregnancy**	62,478 (7.2)		70,614 (43.4)
**Dose 2 of COVID-19 vaccine during pregnancy**	98,977 (11.4)		50,133 (30.8)
**Smoking status**			
Current	152,078 (17.6)	57,780 (18.2)	27,702 (17)
Ex	118,843 (13.7)	43,708 (13.7)	22,070 (13.6)
Never	542,178 (62.6)	196,999 (61.9)	102,486 (63)
**History of cardiovascular and haematological diseases**^a^	9590 (1.1)	3500 (1.1)	1822 (1.1)
**History of hypertension**^a^	84,740 (9.8)	28,661 (9)	17,558 (10.8)
**History of diabetes**^a^	44,549 (5.1)	15,966 (5)	8721 (5.4)
**History of depression**^a^	178,352 (20.6)	64,645 (20.3)	34,429 (21.2)
**History of other conditions**^a^	131,338 (15.2)	54,043 (17)	17,848 (11)

a In the year before pregnancy.

During follow-up, we identified 182,120 adverse pregnancy outcomes as follows: gestational diabetes (n = 66,595), gestational hypertension (n = 24,440), pre-eclampsia (n = 13,655), preterm births (n = 42,850, including n = 7985 very preterm), infants born small for gestational age (n = 32,170), stillbirths (n = 1065) and venous thrombotic events (n = 1345) (Table 2).

**Table 2:** Incidence of adverse pregnancy outcomes by pandemic period, follow-up, total number of events and incidence rate (IR) per 1000 person-years.

Adverse pregnancy outcomes	Overall			Pre-vaccination era			Vaccination era		
	Follow-up (person-years)	N events	IR	Follow-up (person-years)	N events	IR	Follow-up (person-years)	N events	IR
Gestational diabetes	638,641.2	66,595	104.28 (103.49, 105.07)	234,839.1	23,030	98.07 (96.80, 99.34)	119,367.0	13,385	112.13 (110.24, 114.03)
Gestational hypertension	648,841.1	24,440	37.67 (37.20, 38.14)	238,266.5	8705	36.53 (35.77, 37.30)	121,438.3	4935	40.64 (39.51, 41.77)
Pre-eclampsia	649,334.8	13,655	21.03 (20.68, 21.38)	238,435.0	4990	20.93 (20.35, 21.51)	121,538.9	2850	23.45 (22.59, 24.31)
Preterm birth^a^	496,260.9	42,850	86.35 (85.53, 87.16)	184,342.8	17,255	93.6 (92.21, 95.00)	94,892.1	10,090	106.33 (104.27, 108.41)
Very preterm birth^a^	496,261.0	7985	16.09 (15.74, 16.44)	184,342.8	3470	18.82 (18.20, 19.45)	94,892.1	2305	24.29 (23.30, 25.29)
Small for gestational age^a^	496,261.0	32,170	64.82 (64.12, 65.53)	184,342.8	12,215	66.26 (65.09, 67.44)	94,892.1	6360	67.02 (65.38, 68.68)
Stillbirth^a^	469,647.7	1065	2.27 (2.13, 2.40)	174,644.7	395	2.26 (2.04, 2.49)	89,090.0	235	2.64 (2.30, 2.99)
Thrombotic venous event	621,958.8	1345	2.16 (2.05, 2.28)	228,495.2	455	1.99 (1.81, 2.18)	115,535.9	285	2.47 (2.18, 2.76)

a Excluding women without a recorded estimated gestational age at delivery.

**Fig. 1:**
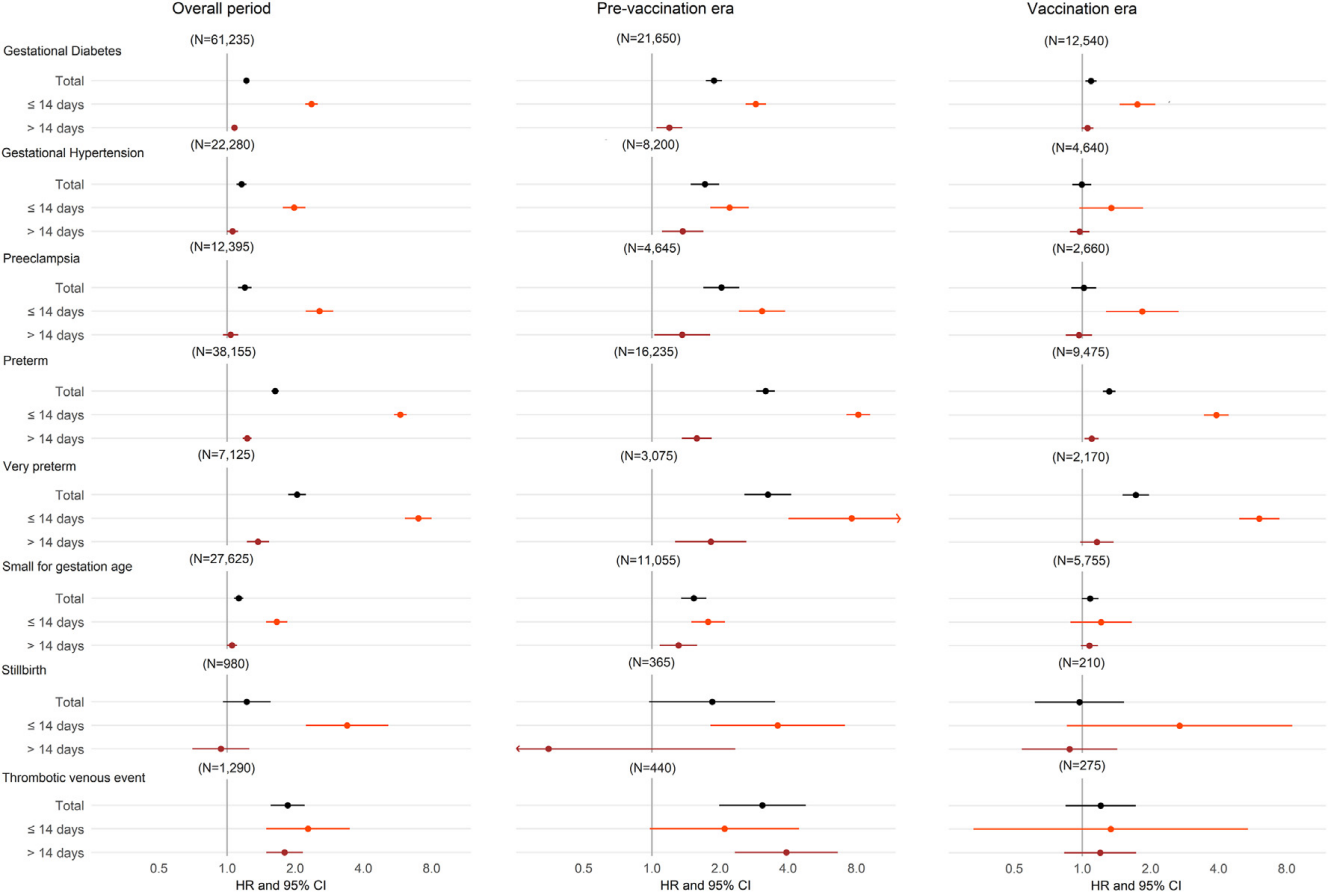
Fully adjusted hazard ratios (log scale) for adverse pregnancy outcomes after COVID-19 diagnosis by pandemic period (overall n = 865,654, prevaccination era n = 318,192, vaccination era n = 162,733).

In the overall pregnancy cohort, COVID-19 diagnosis during pregnancy was associated with a higher risk of gestational diabetes (HR 1.22, 95% CI 1.18–1.26), gestational hypertension (HR 1.16, 1.10–1.22), pre-eclampsia (HR 1.20, 1.12–1.28), preterm birth (HR 1.63, 1.57–1.69; and HR 1.68, 1.61–1.75 for spontaneous preterm), very preterm birth (HR 2.04, 1.86–2.23), small for gestational age (HR 1.12, 1.07–1.18), and thrombotic venous events (HR 1.85, 1.56–2.20) (Fig. 1). Risks were higher within 14 days following COVID-19 diagnosis and remained elevated, although weaker, after 14 days with the exception of pre-eclampsia and small for gestational age. Association with stillbirth was observed only within 14 days following COVID-19 diagnosis (HR 3.39, 2.23–5.15). All associations were stronger in the pre-vaccination era, and remained elevated but weaker during the vaccination era for gestational diabetes (HR 1.09, 1.03–1.16), preterm birth (HR 1.31, 1.23–1.40), very preterm birth (HR 1.72, 1.50–1.97). Associations with hospitalised COVID-19 were generally stronger than associations with non-hospitalized COVID-19 (Figure S2).

**Fig. 2:**
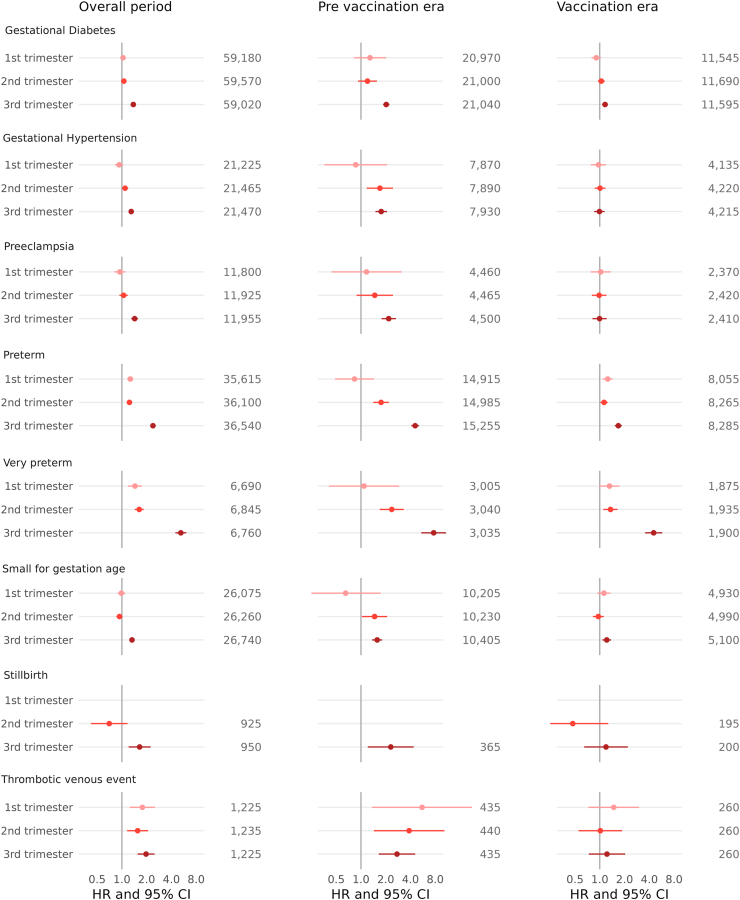
Fully adjusted hazard ratios (log scale) for adverse pregnancy outcomes after COVID-19 diagnosis by pandemic period and trimester of infection (n = 829,180, including only England).

All associations between COVID-19 diagnosis and adverse pregnancy outcomes were confirmed in the 3rd trimester and remained elevated in the 1st and 2nd trimesters for preterm, very preterm, and thrombotic venous events (Fig. 2 and Figure S3). Hazard ratios for associations between COVID-19 diagnosis in the 3rd trimester and adverse pregnancy outcomes were generally stronger in magnitude during the pre-vaccination era, and remained elevated for gestational diabetes, preterm, very preterm birth and small for gestational age during the vaccination era. All associations were stronger in the original variant period, and remained elevated but weaker during the alpha and delta eras for gestational diabetes (Figure S4).

Associations between COVID-19 diagnosis and adverse pregnancy outcomes were weaker in pregnant women who were fully vaccinated compared those who were not fully vaccinated for gestational diabetes, preterm and very preterm birth (Figure S5).

In subgroup analyses, having a previous COVID-19 diagnosis before pregnancy modified the association between COVID-19 diagnosis during pregnancy with gestational hypertension (HRs for women with vs without a previous COVID-19 diagnosis: 1.57 (1.13, 2.20) vs 1.15 (1.09–1.21), p-value_interaction_ = 0.0129) and pre-eclampsia (1.82 (1.19–2.80) vs 1.19 (1.11–1.27), p-value_interaction_ = 0.0029). No significant effect modifiers were observed on the multiplicative scale (Figures S6–S11).

Results remained consistent in sensitivity analyses (Figures S12 and S13).

### COVID-19 vaccination

Among 148,841 pregnant women unvaccinated before pregnancy, 60,875 (40.9%) women were vaccinated with dose 1 of COVID-19 vaccine during pregnancy and 15,680 (10.5%) women were diagnosed with COVID-19 (Table S5). Compared to those vaccinated with dose 1, unvaccinated women were generally younger, more likely to be current smokers, from non-white ethnic groups and resided in areas with higher deprivation level.

COVID-19 vaccination during pregnancy was associated with a lower risk of COVID-19 diagnosis during pregnancy (Figure S14). After dose 1 of COVID-19 vaccine, HRs were 0.70 (0.60–0.81) in the first 4 weeks, 0.66 (0.59–0.73) during week 5–8, and 0.60 (0.58–0.62) from 9 weeks. After dose 2, HRs declined from 0.75 (0.68–0.84) in the first 4 weeks to 0.67 (0.60–0.73) and 0.55 (0.51–0.59) in subsequent periods. We found similar patterns of associations in subgroup and sensitivity analyses (Figures S15 and S16), with the notable exception that there was not a clear benefit of vaccination in pregnant women with a history of COVID-19 diagnosis.

During follow-up, there were 11,920 records of gestational diabetes, 4355 gestational hypertension, 2470 pre-eclampsia, 250 venous thrombotic, 8605 preterm birth (including 1860 very preterm birth), 5830 small for gestational age and 215 stillbirth events (Table S6).

**Fig. 3:**
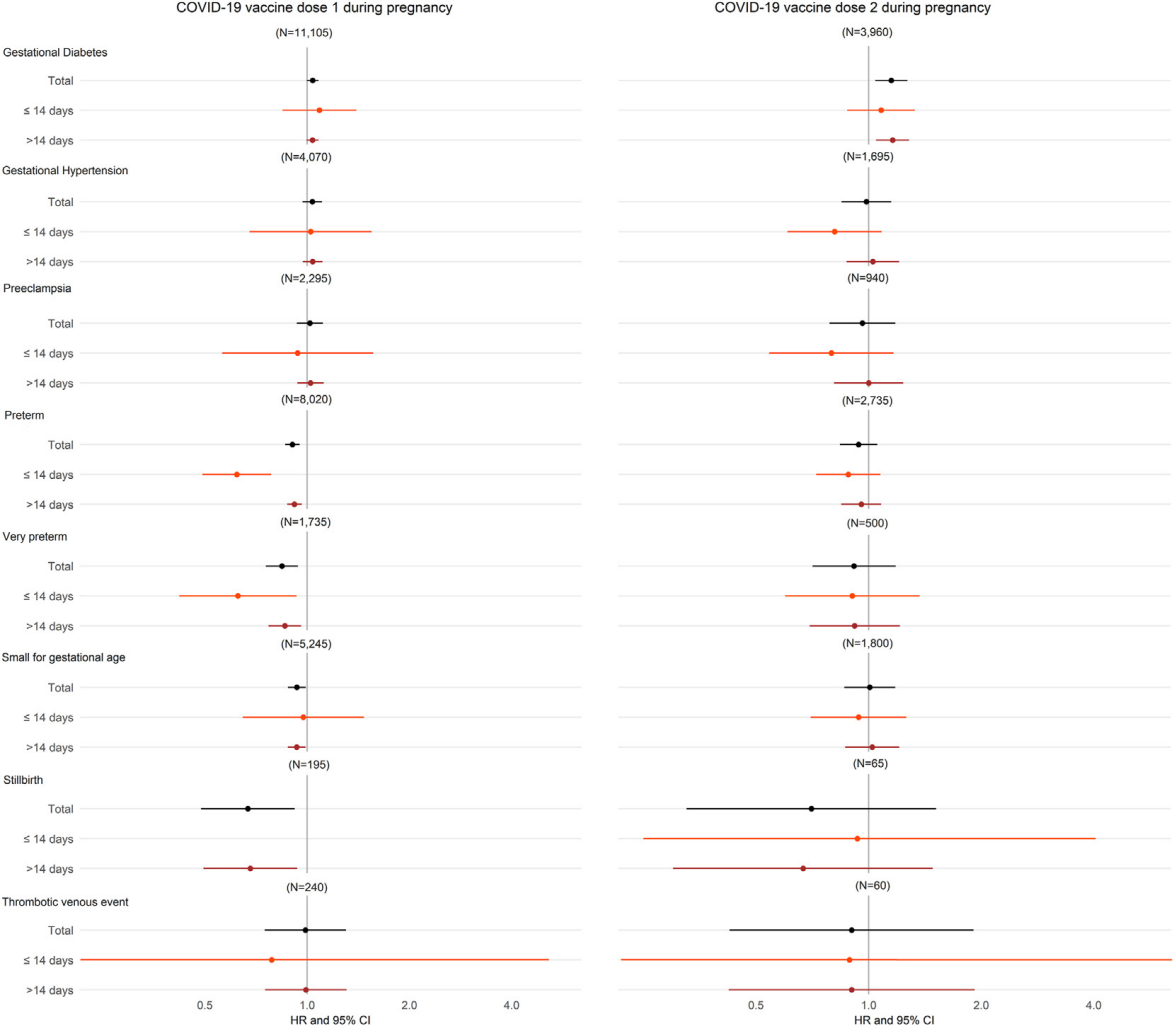
Fully adjusted hazard ratios (log scale) for adverse pregnancy outcomes, after dose 1 and 2 of COVID-19 vaccine during pregnancy (dose 1 analysis n = 148,841, dose 2 analysis n = 57,885, dose 2 analysis includes only England).

Dose 1 of COVID-19 vaccine during pregnancy was associated with lower risks of preterm birth (HR: 0.90, 0.86–0.95), very preterm birth (HR: 0.84, 0.76–0.94), small for gestational age (HR: 0.93, 0.88–0.99), and stillbirth (HR: 0.67, 0.49–0.92) (Fig. 3). However, we did observe a marginal higher incidence of gestational diabetes in women after dose 1 of COVID-19 vaccine during pregnancy in comparison to women without dose 1 of COVID-19 vaccine during pregnancy (HR: 1.04, 1.00–1.08). This association appeared to be restricted to women under 30 years of age or those in the 3rd trimester of pregnancy (Figures S17–S19). Similarly, we observed a slightly higher incidence of gestational diabetes in women after dose 2 of COVID-19 vaccine during pregnancy in comparison to women receiving only dose 1 of COVID-19 vaccine during pregnancy (Fig. 3 and Table S7). Notably, in sensitivity analyses, these associations attenuated to the null upon censoring follow-up at incident COVID-19 diagnosis (Figure S20). There was no evidence to suggest a higher incidence of other adverse pregnancy outcomes after COVID-19 vaccination during pregnancy (Fig. 3), which was consistent across subgroups and in sensitivity analyses (Figures S17–S21).

## Discussion

The main finding of this analysis was that COVID-19 diagnosis during pregnancy was associated with higher risk of common adverse pregnancy outcomes, including gestational diabetes, hypertensive disorders, preterm birth and small for gestational age, as well as rare outcomes including very preterm, preeclampsia, venous thrombosis and stillbirth (only within 14 days after COVID-19 diagnosis). These risks were more pronounced within 14 days of COVID-19 diagnosis and when COVID-19 diagnosis occurred in the 3rd trimester of pregnancy. These risks were less pronounced after the vaccination rollout in England and Wales. There was no evidence to suggest COVID-19 vaccination during pregnancy was associated with higher risks of adverse pregnancy outcomes. Our study's findings are consistent with previous research that indicate associations of COVID-19 diagnosis during pregnancy with subsequent risk of adverse pregnancy outcomes for pre-eclampsia (HR 1.22 vs RRs in prior studies ranging from 1.31 to 1.76^9^^,^^12^^,^^13^^,^^20^), gestational hypertension (HR 1.16 vs RR 1.46),^9^ and preterm birth (HR 1.63 vs RRs in prior studies ranging from 1.15 to 2.17^7^^,^^8^^,^^12^, ^13^, ^14^). This population-wide study is the largest to date and adds novel findings because we were able to quantify how the risks of adverse pregnancy outcomes vary by time since COVID-19, pregnancy trimester, variant era and before and after vaccination roll-outs. Risks of adverse pregnancy outcomes were lower during the vaccination era compared to the pre-vaccination era, as well as during the alpha and delta eras compared to the original variant period. Disentangling the potential role of the vaccination rollout from the emergence of new variants is challenging due to overlapping time periods and also changes in testing strategies between 2020 and 2021, which may influence the severity of reported COVID-19 diagnoses during these periods. Elevated risk of stillbirth was only observed within 14 days of a COVID-19 diagnosis and not beyond, whilst elevated risks for other adverse pregnancy outcomes were highest within 14 days. This may reflect ascertainment bias, whereby women presenting with a pregnancy complication are more likely to have a COVID-19 test, or may reflect an acute effect of COVID-19, which requires further investigation.

Our study contributes towards the understanding of the risk-benefit ratio of COVID-19 vaccination during pregnancy. Specifically, we find that pregnant women who received the vaccine had a lower risk of COVID-19 diagnosis after receiving a COVID-19 vaccine during pregnancy as shown in studies in the general population.^37^^,^^38^ We also observed a lower risk of gestational diabetes, preterm birth, and very preterm birth following COVID-19 in pregnant women who were fully vaccinated compared to those who were not fully vaccinated. Furthermore, we confirm the safety of the vaccine even for rare and severe events such as stillbirth and venous thrombotic events.^39^, ^40^, ^41^ Vaccination was also associated with a lower risk of preterm births, small for gestational age and stillbirth, likely as a result of fewer and less severe COVID-19. Whilst we observed an association between COVID-19 vaccination and gestational diabetes, this attenuated to the null on censoring for COVID-19 and on excluding pregnant women with comorbidities and in priority groups for COVID-19 vaccination, suggesting this may be due to confounding. Specifically, COVID-19 or other comorbidities may increase the likelihood of gestational diabetes and being vaccinated. Alternatively, the positive association may reflect a type 1 error arising from multiple testing or may have been influenced by differences in healthcare utilisation and outcome recording between vaccinated and unvaccinated women. For example, women with better access to care are more likely to receive both the oral glucose tolerance test for gestational diabetes and the COVID-19 vaccination.

There are several key strengths to our study. To limit reverse causality, we focused on COVID-19 diagnosis during pregnancy and compared the magnitude of the associations over the pregnancy and split the follow-up time into 0–14 and 14+ days since COVID-19 diagnosis or vaccination. To limit confounding, we adjusted for several potential confounders using non-linear propensity scores. To examine possible misclassification of COVID-19 diagnosis during the 1st wave of COVID-19 pandemic, we performed analyses separately for the pandemic period and the vaccination era. To minimise possible misclassification of the outcomes, we integrated primary care, secondary care, and maternity data. Our results were robust to a variety of sensitivity analyses, and the national nature of the pregnancy cohorts supported the generalizability of the findings.

Our study has some potential limitations. First, gestational week at birth was imputed for about 25% of women in England. It is likely that missing gestational week is independent of outcomes and does not affect the time between exposure and the outcomes. Missing data could have resulted in non-differential misclassification of exposure early in pregnancy. However, sensitivity analyses among women with known estimated gestational week confirmed the findings. Second, pregnant women diagnosed with COVID-19 may have received more intensive monitoring than those non-diagnosed. This increased surveillance could lead to a more rigorous case ascertainment, potentially biasing associations away from the null. Third, our analyses exclude home deliveries (about 2.5%) in England.^42^ Fourth, we used a stringent definition for small for gestational age, employing the 5th percentile. This helps in identifying the smallest babies, thereby increasing specificity (i.e., the babies we pinpoint are more likely to face health risks). However, this approach might decrease sensitivity (i.e., we could overlook some babies who have health risks due to their size). Fifth, this study cannot exclude residual confounding, particularly since we lacked data on body mass index and instead adjusted for a history of overweight and obesity and measurement errors in smoking behavior may have biased the estimates toward the null, as smoking behavior is negatively associated with the risk of COVID-19 infection.^43^ Sixth, the potential for reverse causation remains due to relative short pregnancy time periods. Seventh, the results from this study are based on the whole populations in England and Wales with singleton birth and include, original, alpha, and delta variant periods (up to 31st December 2021), but may not be generalisable to other countries, non-singleton birth pregnancies or other pandemic periods. Eighth, this study cannot exclude the possibility of effect modification on scales other than multiplicative ones.

In conclusion, our study provides evidence that COVID-19 diagnosis during pregnancy was associated with a higher risk of common and rare adverse pregnancy outcomes. These risks were more pronounced within 14 days of COVID-19 diagnosis and when COVID-19 diagnosis occurred in the 3rd trimester of pregnancy, and before the vaccination rollout. Notably, there was no evidence to suggest higher risks of adverse pregnancy outcomes following vaccination. These findings support recommendations towards high-priority vaccination against COVID-19 in pregnant women to avoid high risks of adverse pregnancy outcomes from COVID-19 especially in the 3rd trimester.

## Contributors

ER, TB, GS, AA, KH, GC, DAL, MC, AMW contributed to Conceptualization. ER, TB, JN, LZ, RD, EA, AA, DAL, AMW contributed to Methodology. ER contributed to Formal analysis. ER, AMW contributed to Investigation. ER, TB, JN, HA contributed to Data Curation. ER, AMW contributed to Writing–Original Draft. All authors contributed to Writing–Review & Editing. ER contributed to Visualization. AMW contributed to Supervision.

## Data sharing statement

The data used in this study are available in NHS England's Secure Data Environment (SDE) service for England, but as restrictions apply they are not publicly available (https://digital.nhs.uk/services/secure-data-environment-service). The CVD-COVID-UK/COVID-IMPACT programme led by the BHF Data Science Centre (https://bhfdatasciencecentre.org/) received approval to access data in NHS England's SDE service for England from the Independent Group Advising on the Release of Data (IGARD) (https://digital.nhs.uk/about-nhs-digital/corporate-information-and-documents/independent-group-advising-on-the-release-of-data) via an application made in the Data Access Request Service (DARS) Online system (ref. DARS-NIC-381078-Y9C5K) (https://digital.nhs.uk/services/data-access-request-service-dars/dars-products-and-services). The CVD-COVID-UK/COVID-IMPACT Approvals & Oversight Board (https://bhfdatasciencecentre.org/areas/cvd-covid-uk-covid-impact/) subsequently granted approval to this project to access the data within NHS England's SDE service for England. The de-identified data used in this study were made available to accredited researchers only. Those wishing to gain access to the data should contact bhfdsc@hdruk.ac.uk in the first instance.

## Declaration of interests

G Smith: GSK. Consultant and member of expert panel for RSV vaccination in pregnancy. GSK. Member of Data Safety Monitoring Committee for trials of RSV vaccination in pregnancy Moderna. Member of Data Safety Monitoring Committee for trials of RSV vaccination in pregnancy. No other conflicts of interest to be disclosed. D A Lawlor: European Research Council, Research grant administered by University of Bristol. No conflict with this paper; and Diabetes UK, Research grant administered by University of Bristol. No conflict with this paper.
