# Supplementary material for: COVID-19 diagnosis, vaccination during pregnancy, and adverse pregnancy outcomes of 865,654 women in England and Wales: a population-based cohort study
Source: Lancet Reg Health Eur. 2024 Aug 22;45:101037. doi: 10.1016/j.lanepe.2024.101037 (PMC11388188; doi:10.1016/j.lanepe.2024.101037)
Supplement: Supplementary Methods, Figures, and Tables [file mmc1.pdf]

SUPPLEMENTARY METHODS

Data curation and pregnancy cohort definition

The data curation pipeline was developed as consistent as possible to create a research ready dataset allowing to run almost the same analytical code in NHS England's Secure Data Environment (SDE) service for England and the SAIL Databank.

At the beginning of the pipeline we established a master notebook which included all the notebooks involved in the data curation pipeline, along with their respective paths. A parameter notebook was also created, acting as a cornerstone for the entire project. It contained essential details such as data sources names, paths to functions/datasets/project folder, study population start and end dates, latest available batches for archive data tables.

We defined the pregnancy cohort including women registered with primary care services and had a hospital delivery episode, a 1st estimated pregnancy start date from August 1st 2019 and delivery date up to December 31st 2021. Information on the delivery episode and the related date were obtained from the maternity tail of the Hospital Episode Statistics Admitted Patient Care (HES APC) for England, as well as from the birth section of the Maternity Indicators Data Set (MIDS) for the SAIL Databank. Both sources include an indicator that provides details on the reason for access to care. Estimated pregnancy start date was determined as: 'delivery date' – ('gestational week at delivery'*7 days). Converting weeks into days may introduce some inaccuracies. Gestational week was derived from HES APC for England, birth and initial assessment sections of MIDS with preference given to birth section for Wales. In case of data missingness we assumed a typical pregnancy duration of 280 days (n=200,020, 199,145 in England and 875 in Wales) in both SDE and SAIL Databank. The median pregnancy duration (follow-up time) and interquartile range were 280.0 days (273–280) in England and 273 days (266–280.0) in Wales.

Age, sex, and ethnicity were derived from the most recent non-missing value across primary care and secondary care, with preference given to primary care in the event of a match on the same date. Ethnic groups were categorised as "Black or Black British", "Asian or Asian British", "White", "Mixed", "Ethnic minorities" (Other) and "Unknown ethnic group" and further categorised for adjusting as "White", "Other ethnic groups" and "Unknown ethnic group". Ethnic groups were based on Office for National Statistics classification (*Black or Black British*: African, Caribbean, Any other Black/African/Caribbean background. *Asian or Asian British*: Indian, Pakistani, Bangladeshi, Chinese, Any other Asian background. *White*: English/Welsh/Scottish/Northern Irish/British, Irish, Gypsy or Irish Traveller, Any other White background. *Mixed*: White and Black Caribbean, White and Black African, White and Asian, Any other Mixed/Multiple ethnic background. *Ethnic minorities*: Arab, Any other ethnic group).

Socioeconomic deprivation information was derived using the Index of Multiple Deprivation for year 2019. This index measures relative levels of deprivation in 32,844 (England) and 1,909 (Wales) small areas or neighbourhoods, called Lower-layer Super Output Areas (LSOA). Quintile 1 denotes the most deprived; quintile 5 denotes the least deprived.

Exposures, covariates, and outcomes, were defined using code lists based on 5 coding terminologies (International Classification of Diseases 10th Revision - ICD-10, SNOMED Clinical Terms, Read codes, OPCS-4 statistical classifications, Dictionary of medicines and devices - DM+D) and reported in GitHub (>50 phenotypes, and >13000 codes).

The following quality assurance rules were applied: 1) Remove women who are missing sex, year of birth; 2) Remove women whose year of birth is after their year of death; 3) Remove women whose date of death is invalid; 4) Remove women whose date of death is after the death date that was registered in primary care; 5) Remove women whose records contain prostate cancer codes; 6) Remove women who have missing date fields for all primary care records; 7) Remove women with length of gestation less than 12 weeks.

Sample size justification
The sample size in this study included all pregnant women who gave birth in hospitals in England and Wales during the study period and were registered in a primary care practice.

Time varying exposures
Based on previous studies (e.g. Knight et al. 2022), we hypothesised that associations between COVID-19 diagnosis and adverse pregnancy outcomes would be strongest immediately after a COVID-19 diagnosis, and then decline with time since diagnosis (i.e., non-proportional hazards). We therefore calculated hazard ratios in separate time periods (in days: [0,14), [14+,]) after diagnosis of COVID-19, selected to ensure adequate numbers of adverse outcomes each time periods.

Adjustment for propensity score
To enhance efficiency in the estimation of magnitude of the associations, we incorporated a propensity score allowing for more flexible control of confounding;
Specifically, we calculated the propensity score as the conditional probability of exposure, given the observed covariates, by using logistic regression models. To ensure model fitting and capture any nonlinear relationships between the propensity score and the outcome, we applied a spline transformation to the propensity score (3 knots) a necessary adjustment due to the high number of covariates (ethnic group (considering three categories for adjustment: White, Other ethnic groups, Unknown ethnic group), previous pregnancy (yes/no), smoking status (smoker, former smoker, non-smoker), history of hypertension (yes/no), history of diabetes (yes/no), history of haematological and cardiovascular diseases (yes/no), overweight/obesity (yes/no), history of depression (yes/no) and other conditions (yes/no, including at least one of chronic obstructive pulmonary disease, liver disease, chronic kidney disease, cancer and surgical intervention). The models were further adjusted for age (continuous + quadratic term), deprivation index (quintile), calendar week (as a spline term with 3 knots), region in England.

Distribution of the probability of having COVID-19 diagnosis in COVID-19 cases and non-cases in England.

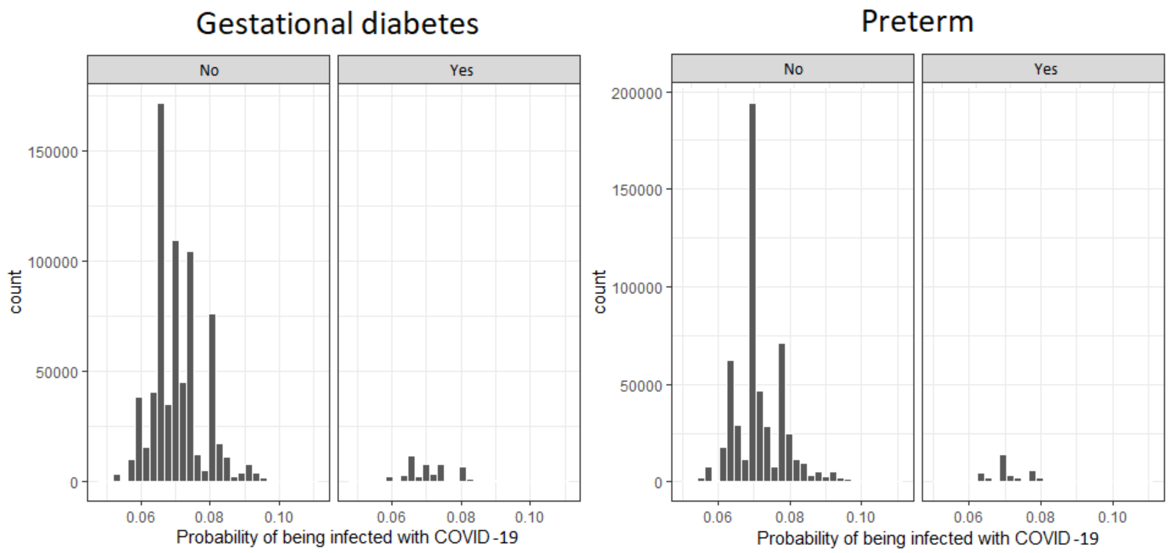

For the overall period and common outcomes (i.e., gestational diabetes and preterm birth) in England, we compared estimates adjusted with the propensity score approach to those from a traditional approach, which incorporated all potential confounders into the model. There was robust consistency between the two analytical methods: HR= 1.22 (95% CI: 1.18-1.26) with both method for gestational diabetes and HR=1.63 (1.57-1.69) using propensity score adjustment vs 1.64 (1.57-1.70) using covariate adjustment for preterm birth.

Log-Log Survival Curves

Example of log-Log Survival Curves to check proportional hazards assumption for pregnant women with (red dotted line) and without COVID-19 diagnosis (blue solid line) within and after 14 days following COVID-19 diagnosis in England.

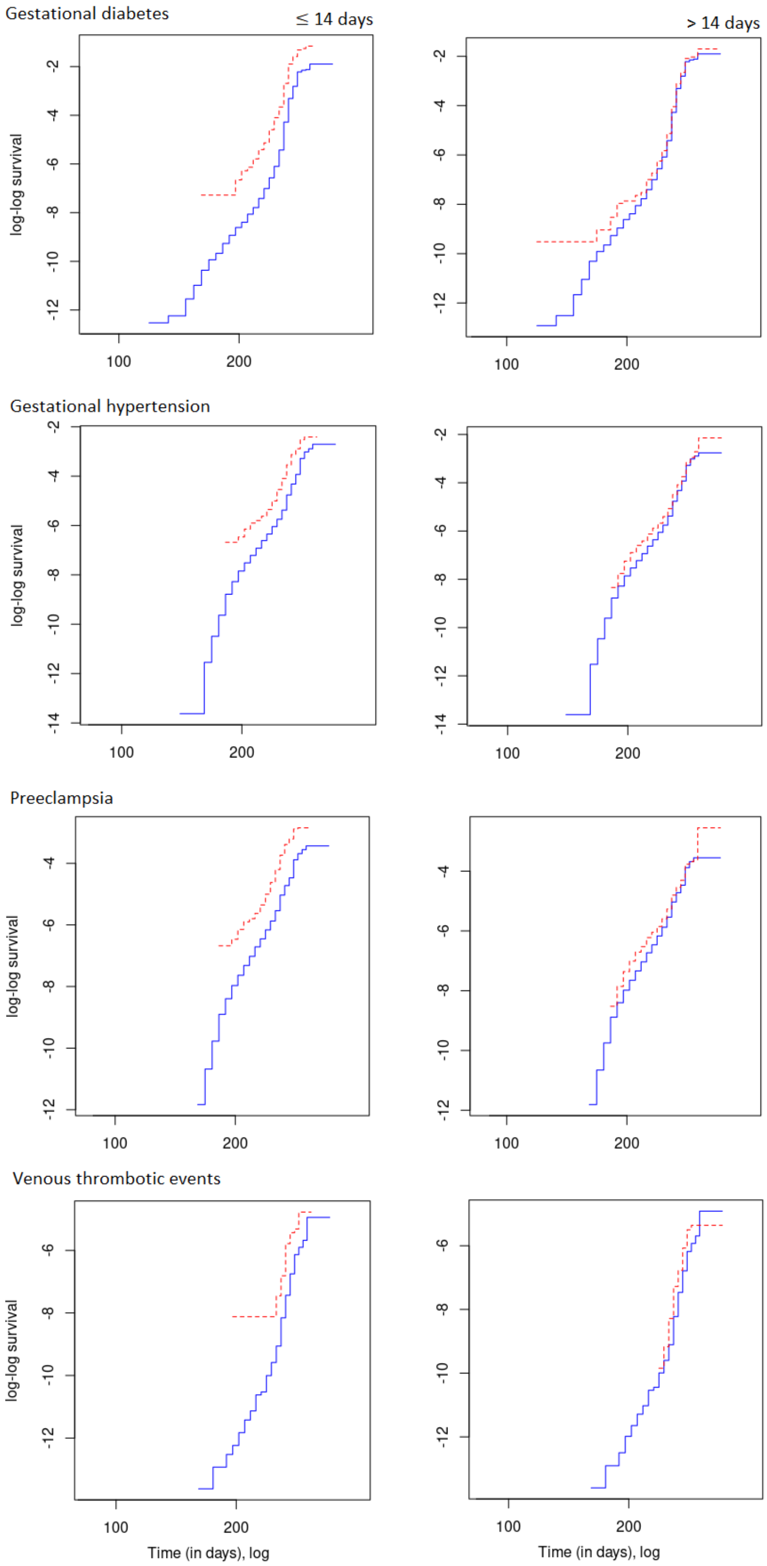

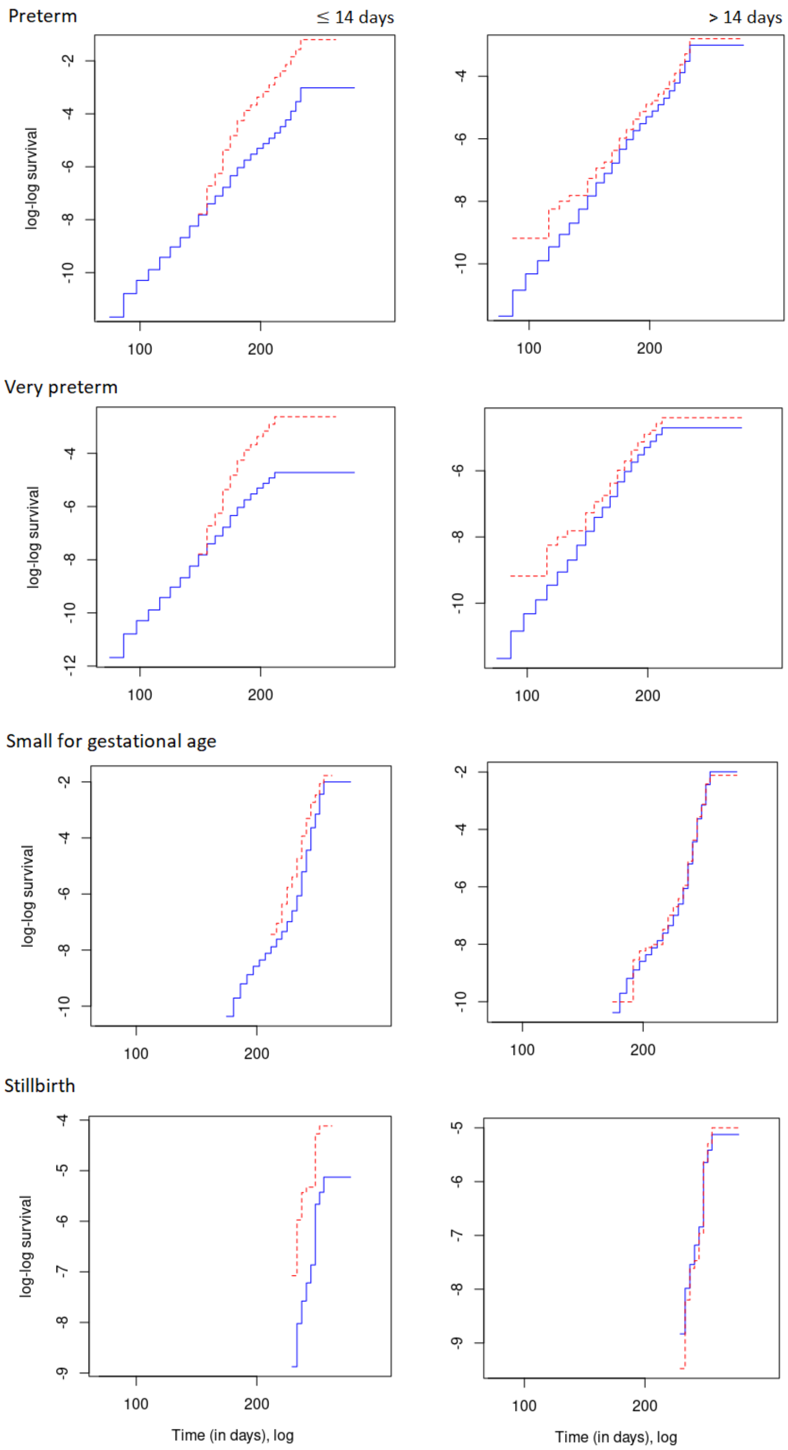
Preterm
≤ 14 days
> 14 days
log-log survival
Very preterm
Small for gestational age
Stillbirth
100
200
Time (in days), log

References

ONS 2021 https://www.ons.gov.uk/methodology/classificationsandstandards/measuringequality/ethnicgroupnationalidentityandreligion#ethnic-group).

Knight et al. 2022. Association of COVID-19 with major arterial and venous thrombotic diseases: a population-wide cohort study of 48 million adults in England and Wales. Circulation 146.12: 892-906.

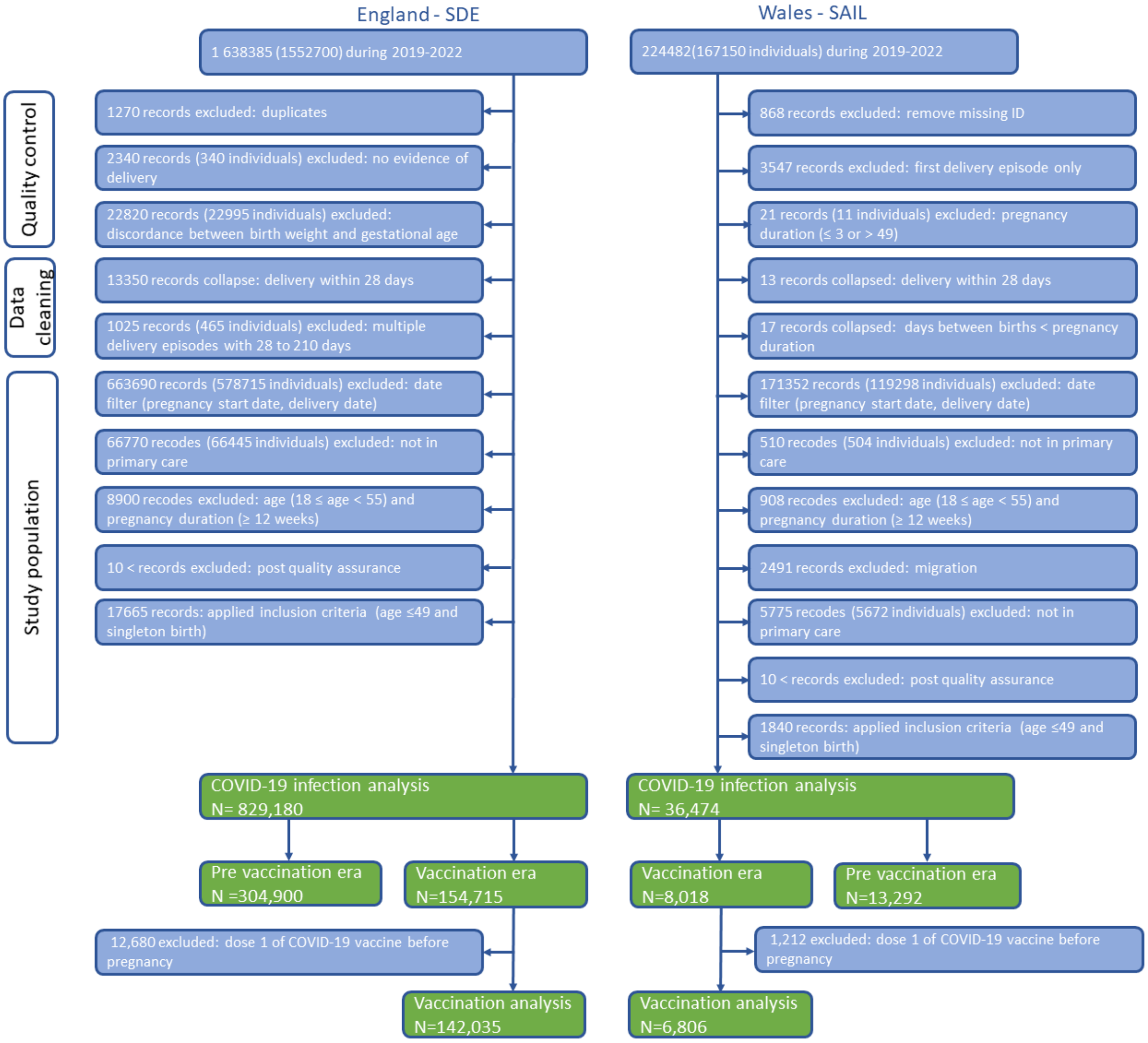

Figure S1. Flow chart - derivation of the pregnancy cohort

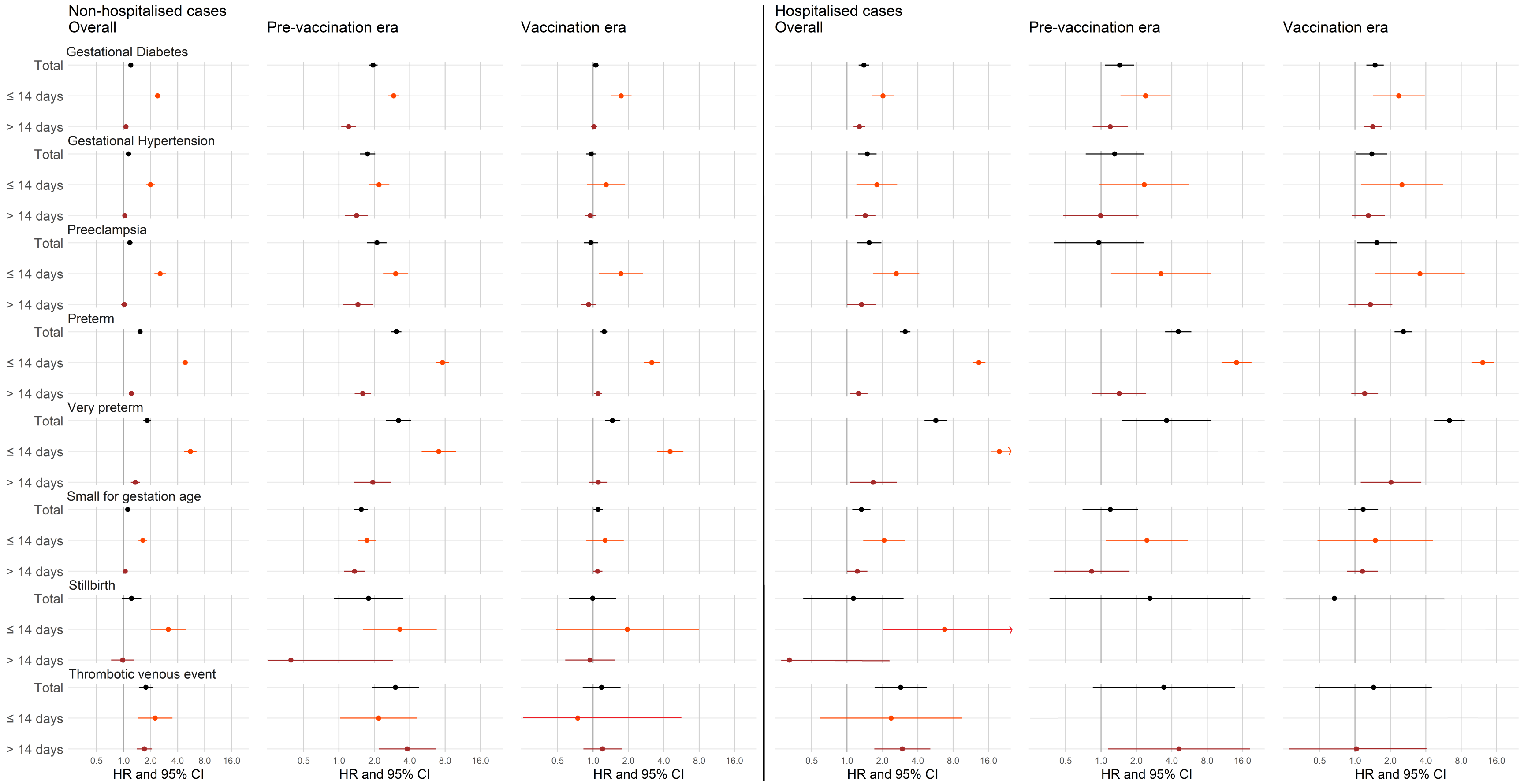

Figure S2. Fully adjusted hazard ratios (log scale) for adverse pregnancy outcomes after COVID-19 diagnosis by pandemic period. The following sensitivity analyses were performed including: non-hospitalized COVID-19 diagnosis (overall period n=829,180, pre-vaccination era n=304,900, vaccination era n=154,715); hospitalized COVID-19 diagnosis (overall period n=829,180, pre-vaccination era n=304,900, vaccination era n=154,715))

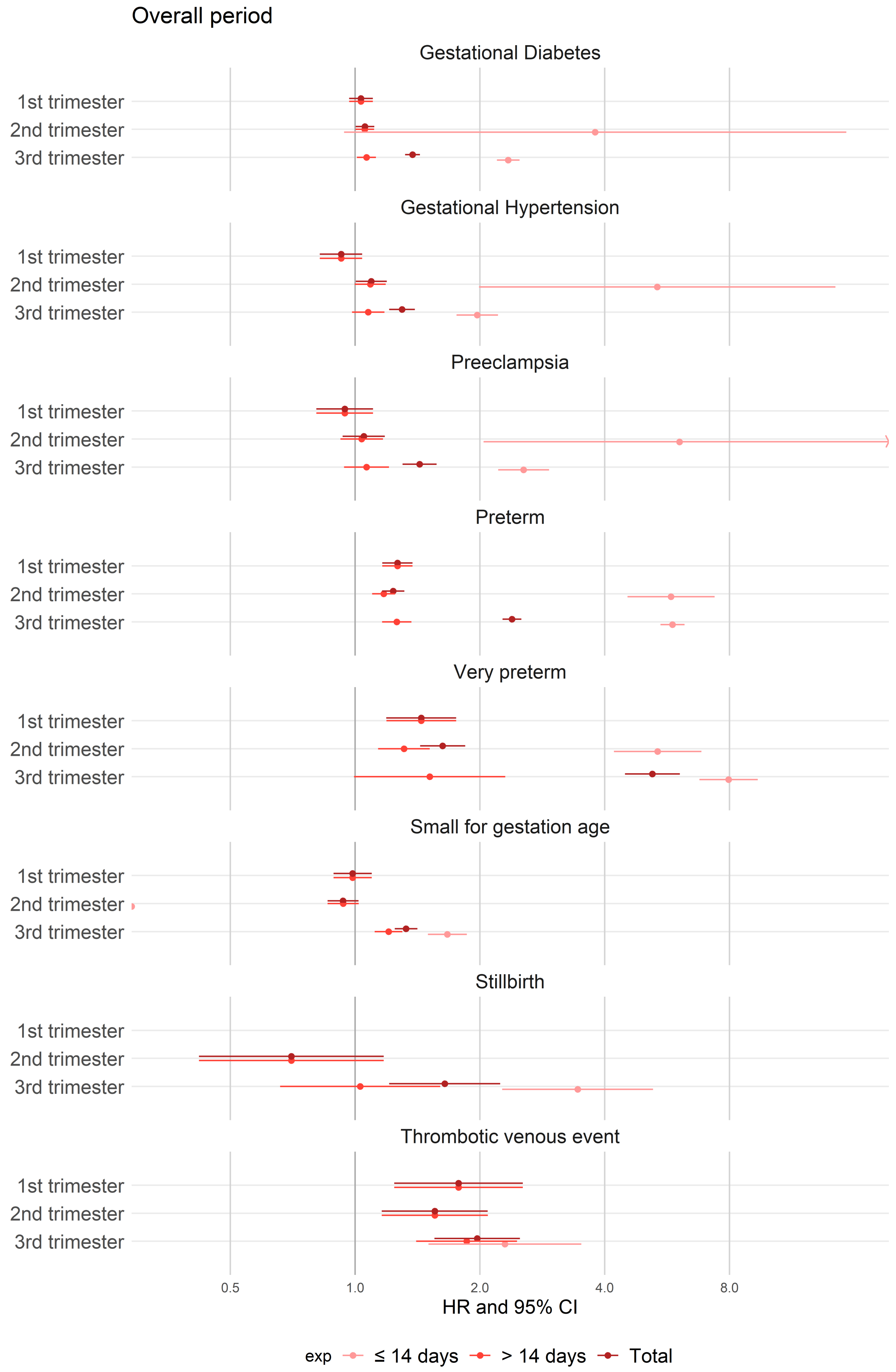

Figure S3. Fully adjusted hazard ratios (log scale) for adverse pregnancy outcomes after COVID-19 diagnosis by trimester (n=829,180)

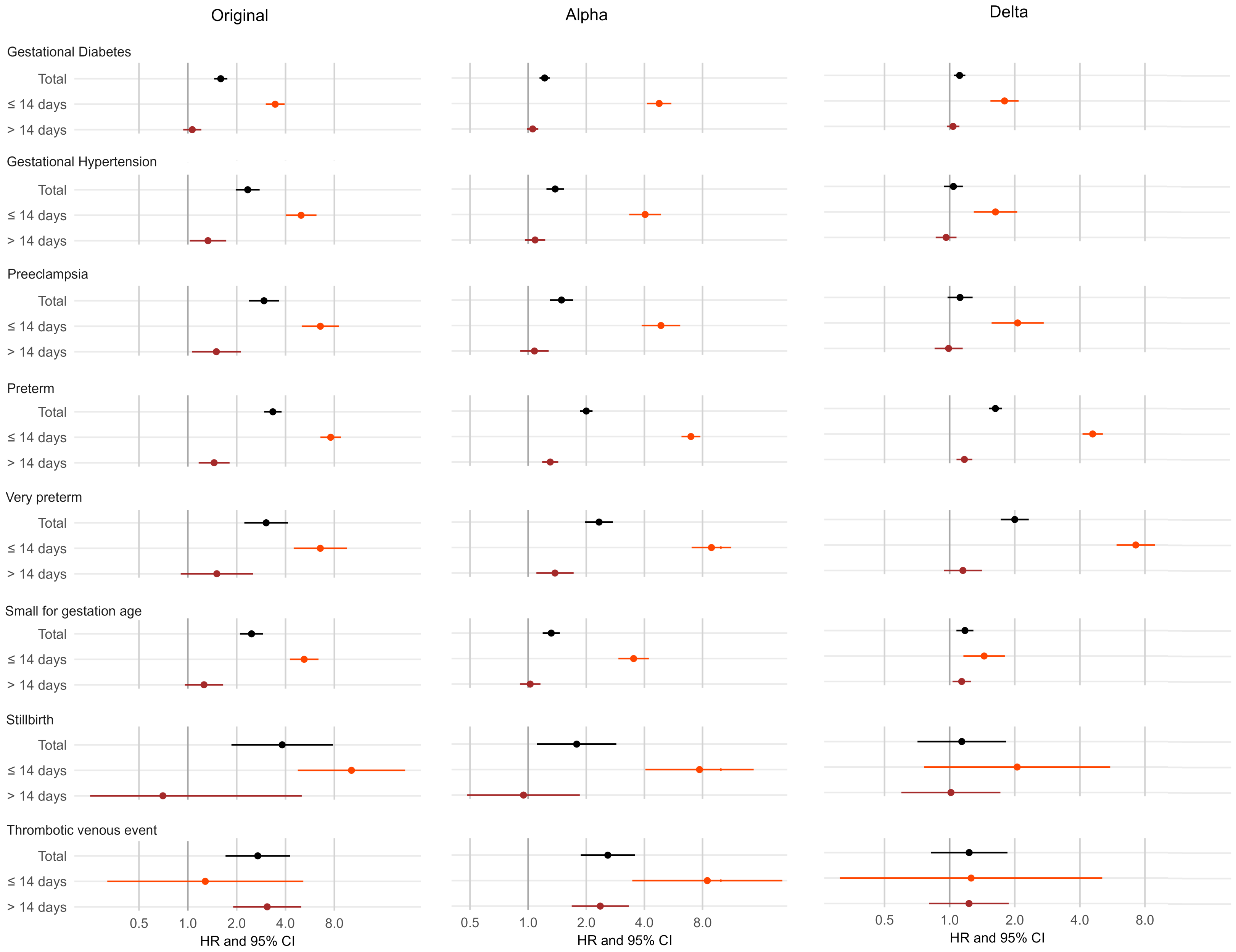

Figure S4. Fully adjusted hazard ratios (log scale) for adverse pregnancy outcomes after COVID-19 diagnosis by variant era (original variant n=591,010; alpha era n=523,880; delta era n=312,290)

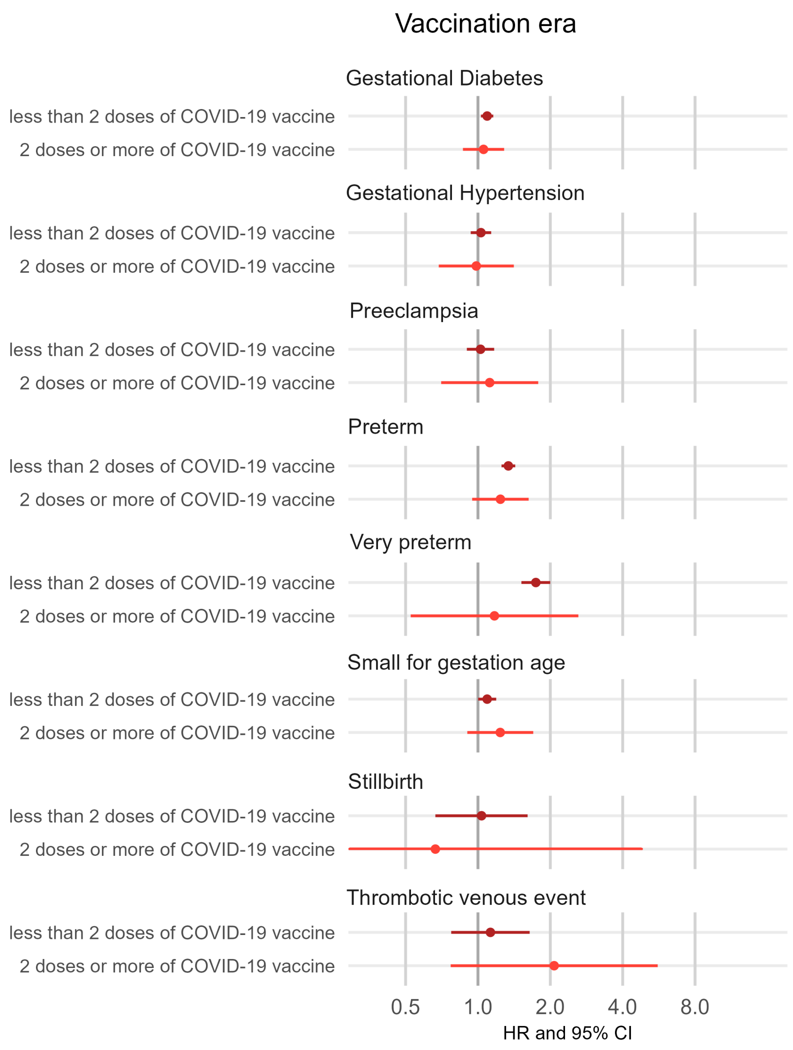

Figure S5. Fully adjusted hazard ratios (log scale) for adverse pregnancy outcomes after COVID-19 diagnosis by COVID-19 vaccination status (vaccination era, n=154,715)

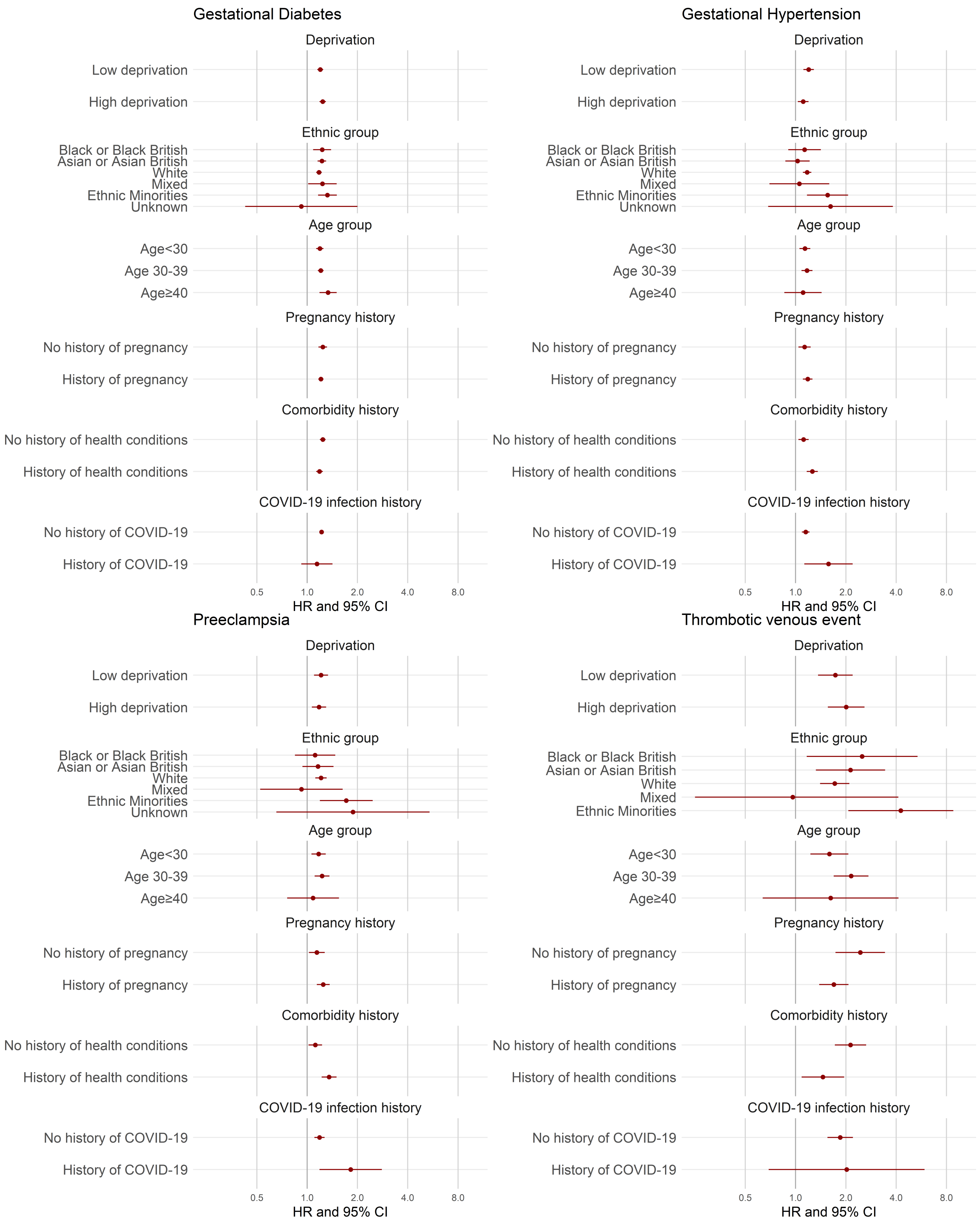

Figure S6. Fully adjusted hazard ratios (log scale) for adverse outcomes during pregnancy after COVID-19 diagnosis by subgroups – overall pandemic period (n=829,180)

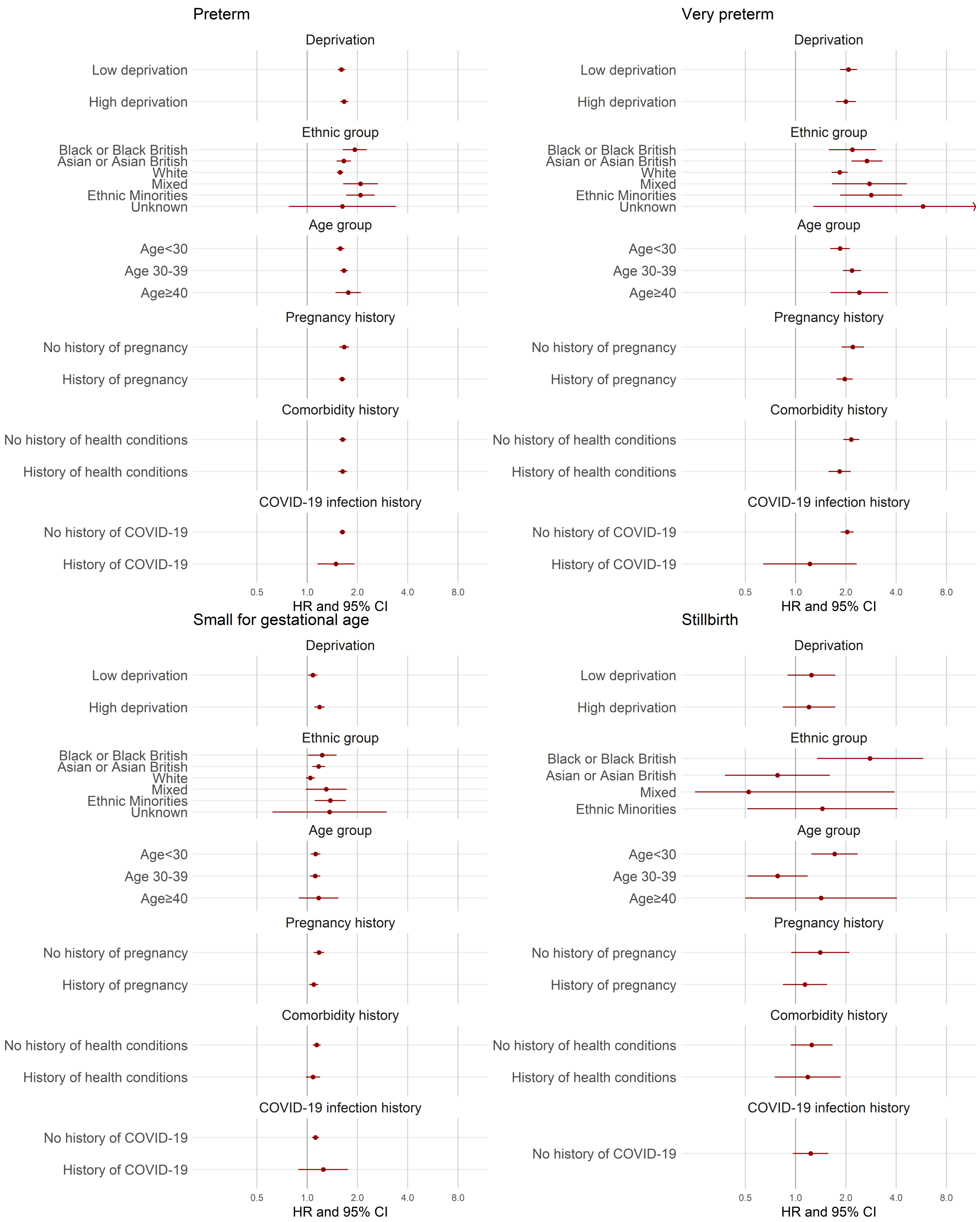

Figure S7. Fully adjusted hazard ratios (log scale) for adverse outcomes at birth after COVID-19 diagnosis by subgroups – overall pandemic period (n=630,035)

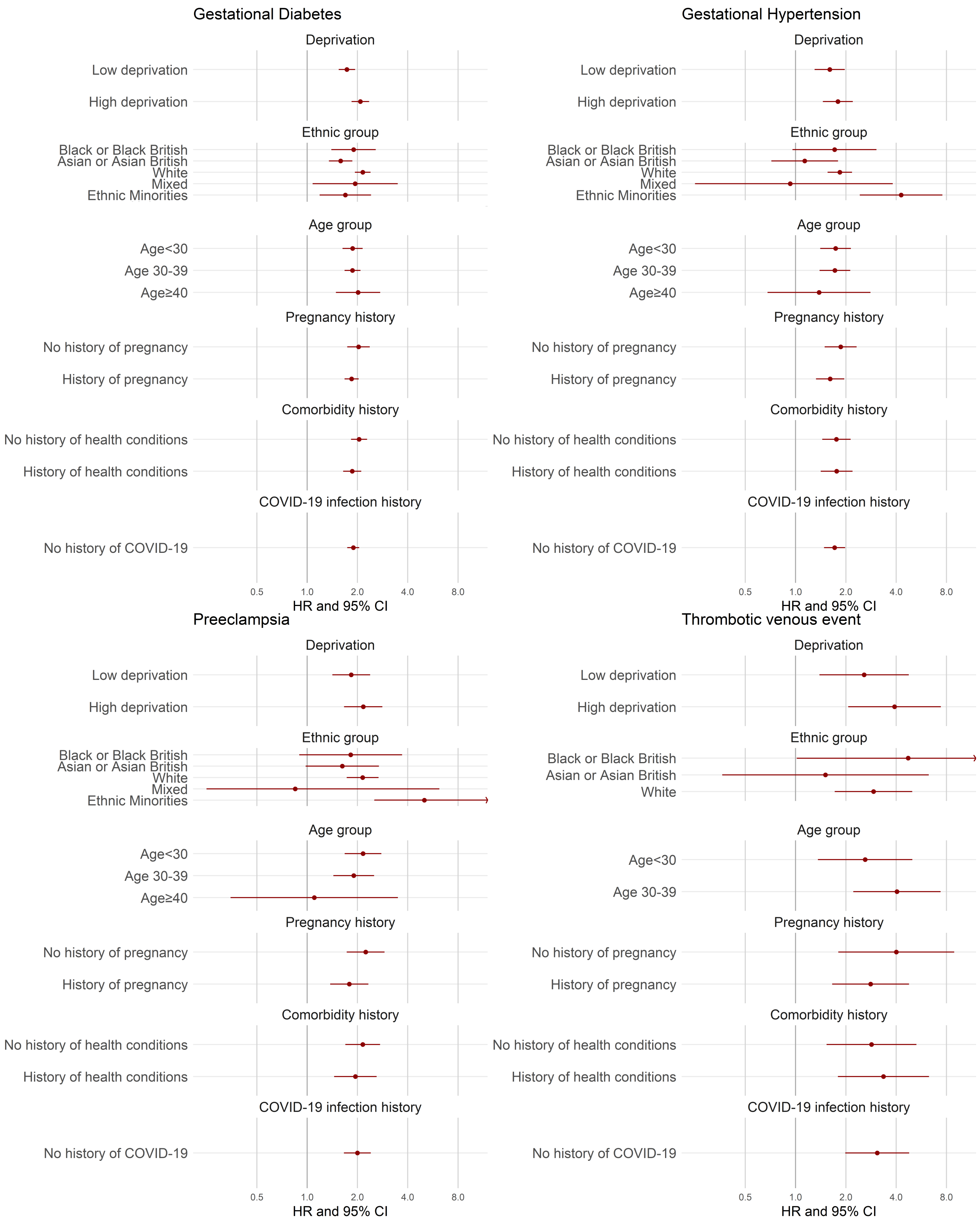

Figure S8. Fully adjusted hazard ratios (log scale) for adverse outcomes during pregnancy after COVID-19 diagnosis by subgroups – pre-vaccination era (n=304,900)

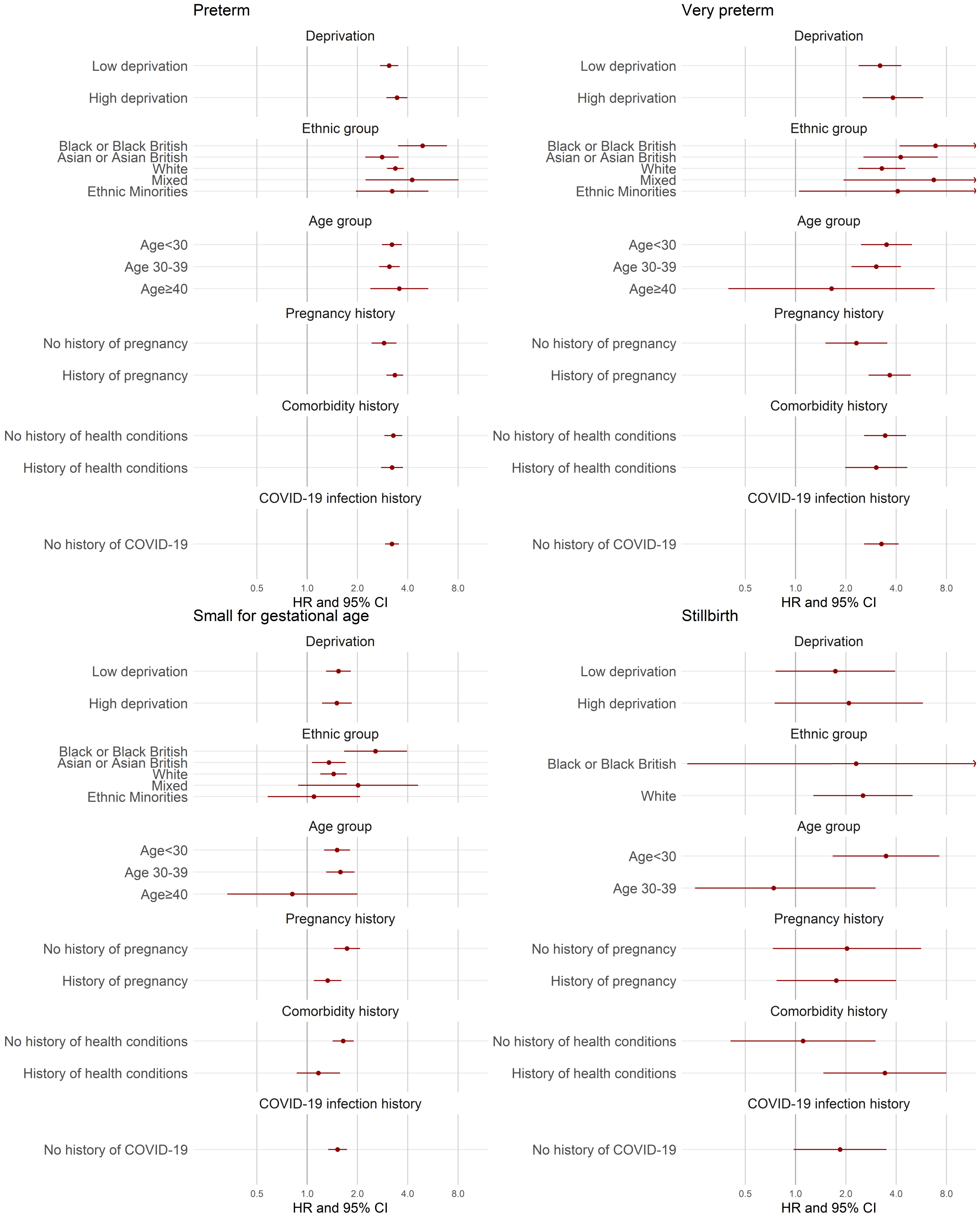

Figure S9. Fully adjusted hazard ratios (log scale) for adverse outcomes at birth after COVID-19 diagnosis by subgroups – pre-vaccination era (n=234,505)

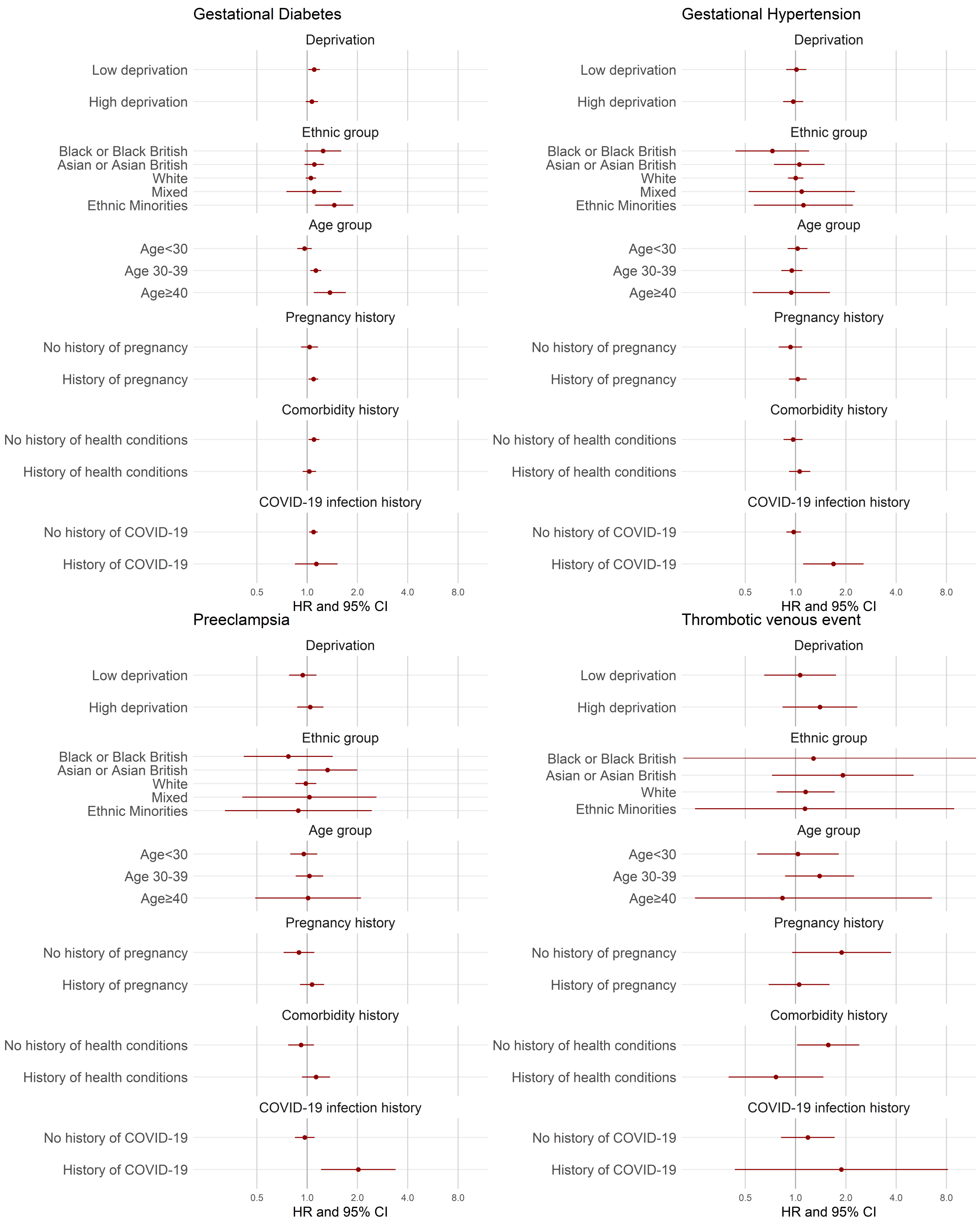

Figure S10. Fully adjusted hazard ratios (log scale) for adverse outcomes during pregnancy after COVID-19 diagnosis by subgroups – vaccination era (n=154,715)

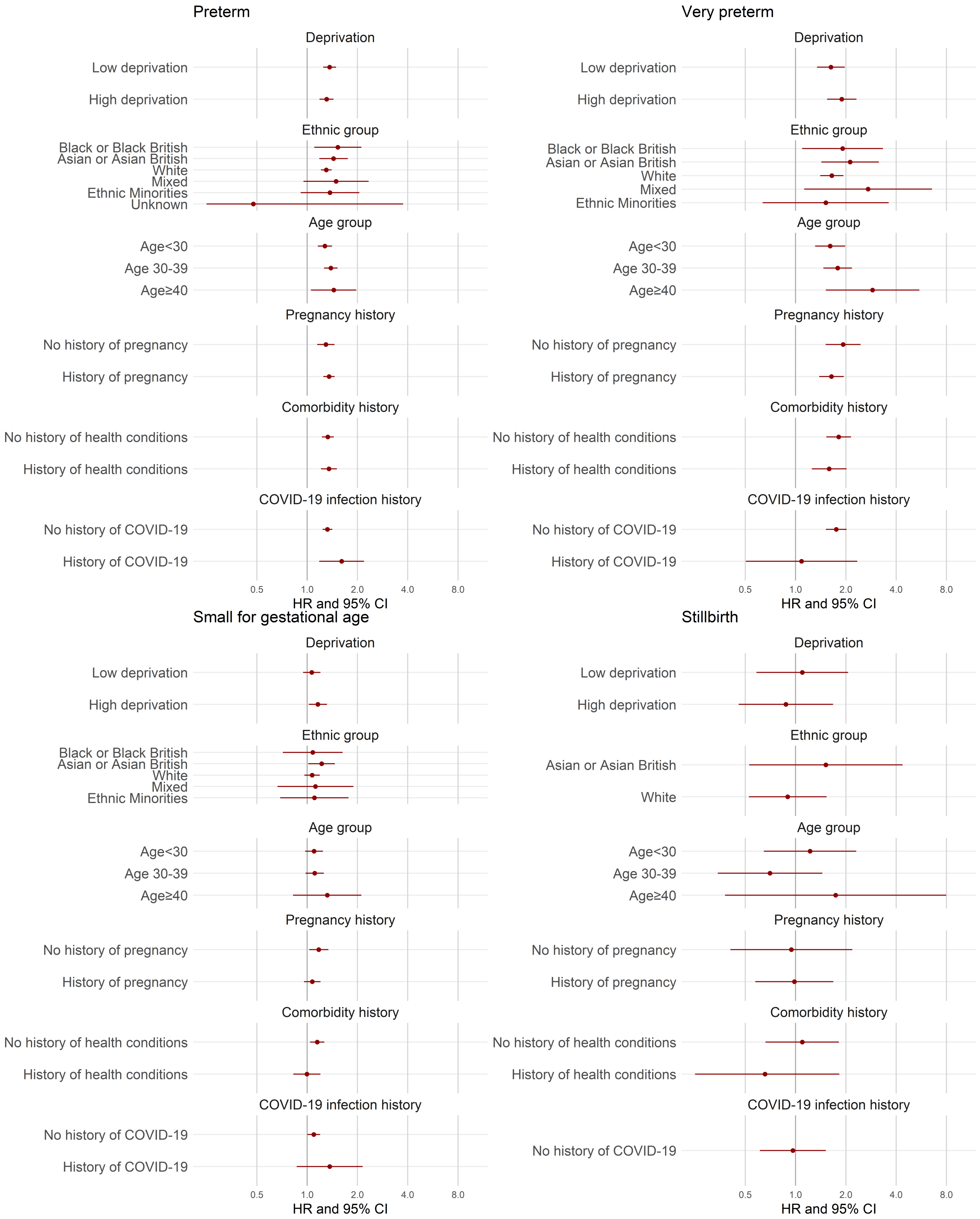

Figure S11. Fully adjusted hazard ratios (log scale) for adverse outcomes at birth after COVID-19 diagnosis by subgroups – vaccination era (n=120,120)

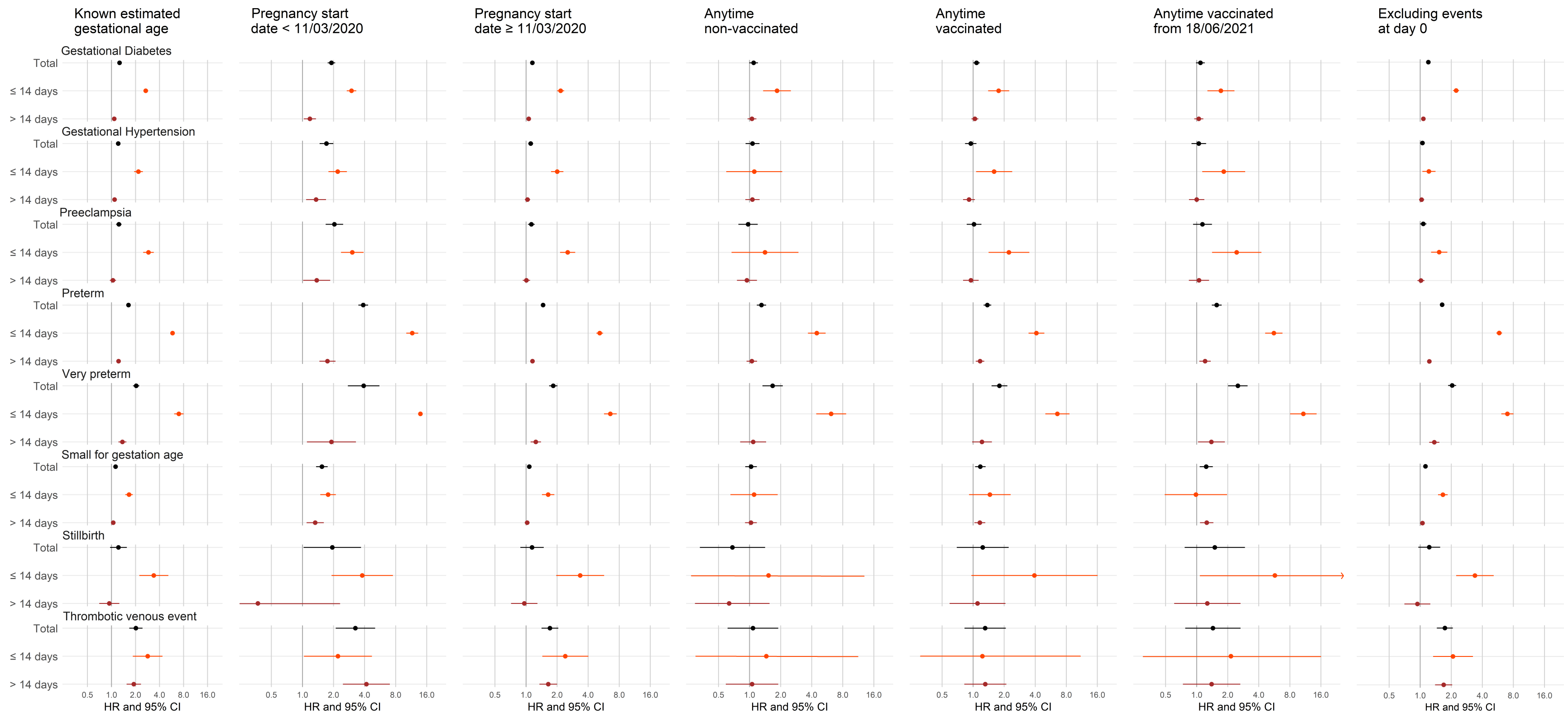

Figure S12. Fully adjusted hazard ratios (log scale) for adverse pregnancy outcomes after COVID-19 diagnosis by pandemic period. The following sensitivity analyses were performed including: (1) women with known estimated gestation age (n=630,035); (2) women with estimated pregnancy start date before March 11th 2020 (n=307,515); (3) women with estimated pregnancy start date after March 11th 2020 (n=521,660); (4) women who had not received any doses of COVID-19 vaccine up to December 31st 2021 (n=58,005), (5) women who had received at least one dose of COVID-19 vaccine up to December 31st 2021 (n=96,710) and (6) women who had received at least one dose of COVID-19 vaccine from 18th of June 2021 to December 31st 2021 (n=53,080), (7) outcomes that occurred from day 1 of the follow-up)

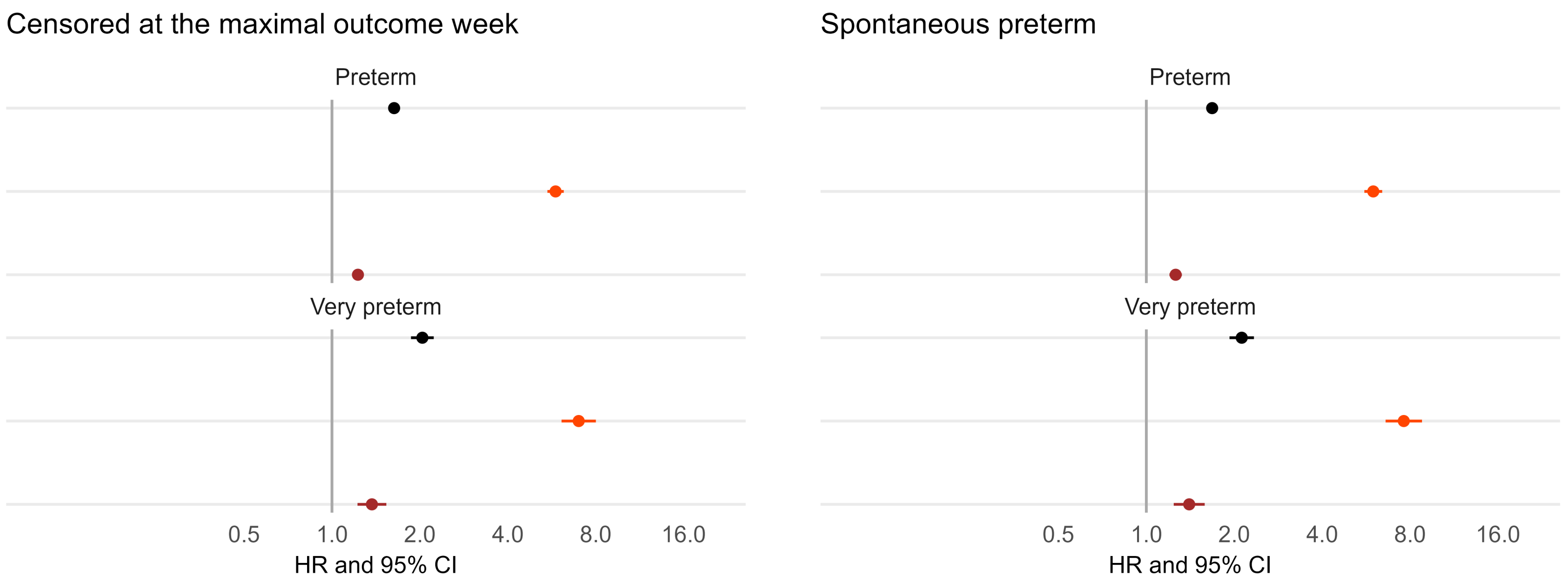

Figure S13. Fully adjusted hazard ratios (log scale) for adverse pregnancy outcomes after COVID-19 diagnosis. The following sensitivity analyses were performed censoring follow-up to the maximal outcome week and including spontaneous preterm as outcome of interest (n=829,180)

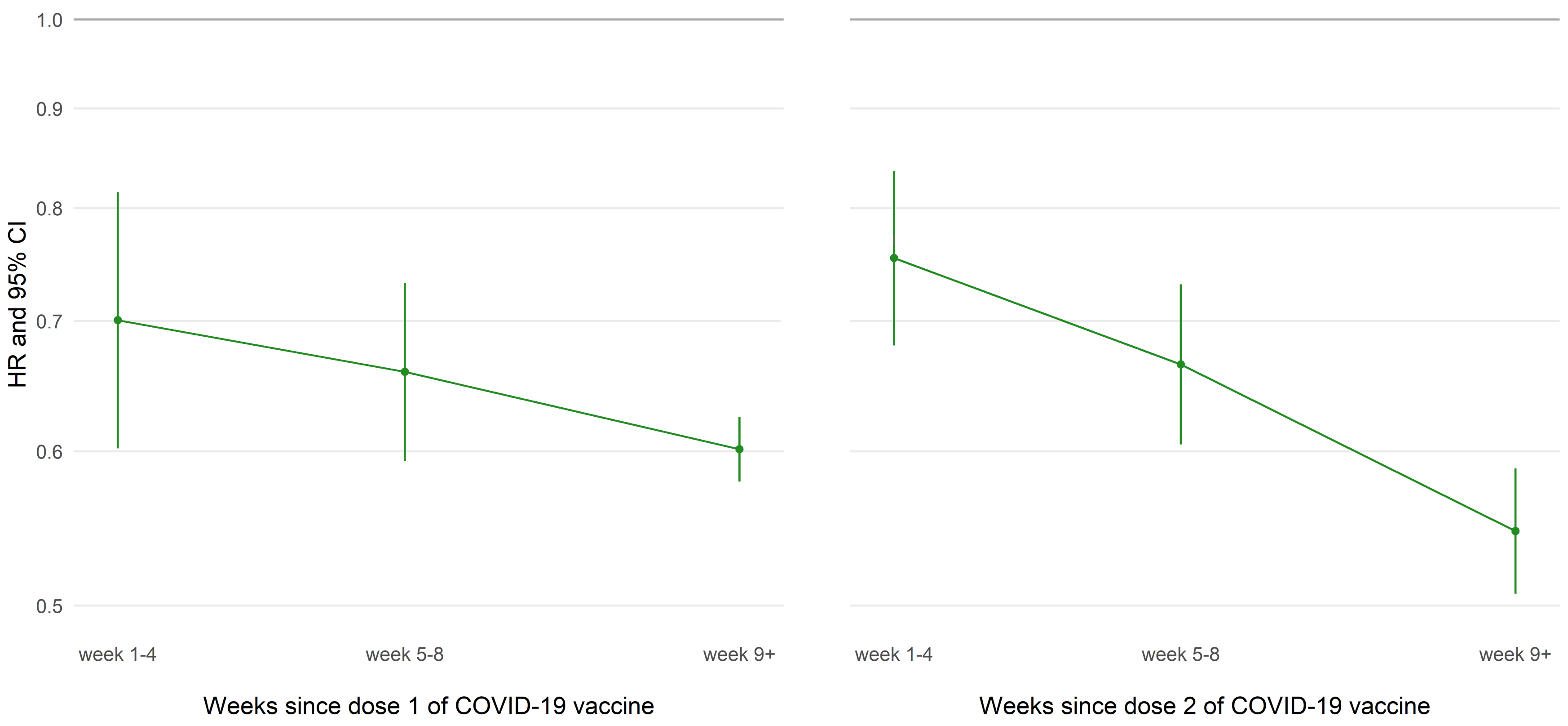

Figure S14. Fully adjusted hazard ratios (log scale) for COVID-19 diagnosis after dose 1 and 2 of COVID-19 vaccine by time since vaccination (dose 1 analysis n= 148,841, dose 2 analysis n= 57,885, dose 1 analysis includes England and Wales and dose 2 analysis includes only England

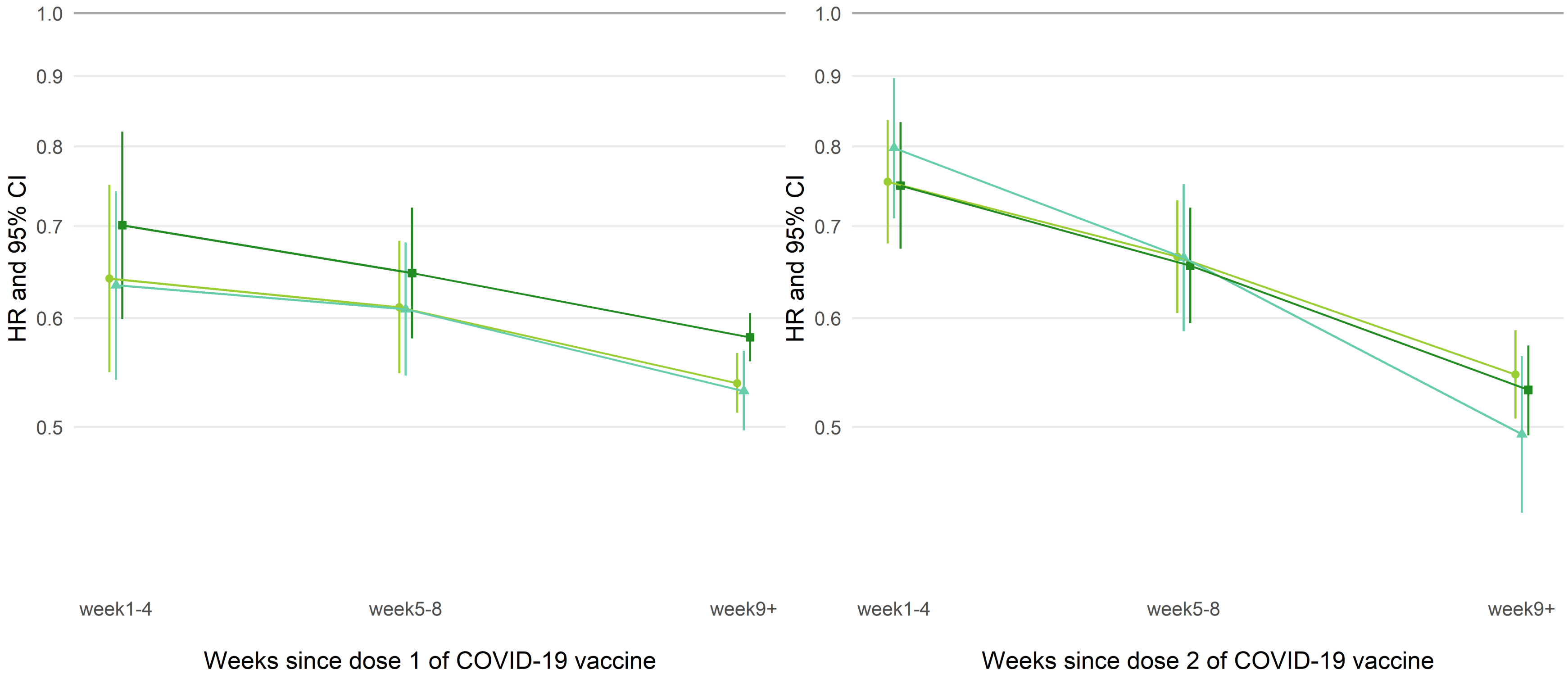

Figure S15. Fully adjusted hazard ratios (log scale) for COVID-19 diagnosis after dose 1 and 2 of COVID-19 vaccine during pregnancy. Sensitivity analyses involved: restricted the analyses to: women without previous COVID-19 diagnosis (dark green, n=131,845 and n=54,240 for dose 2), women anytime vaccinated (yellow-green, n=84,025 for dose 1 and n=57,885 for dose 2) and women anytime vaccinated from 18th of June 2021 (turquoise-green, n=53,060 and n=26,935 for dose 2

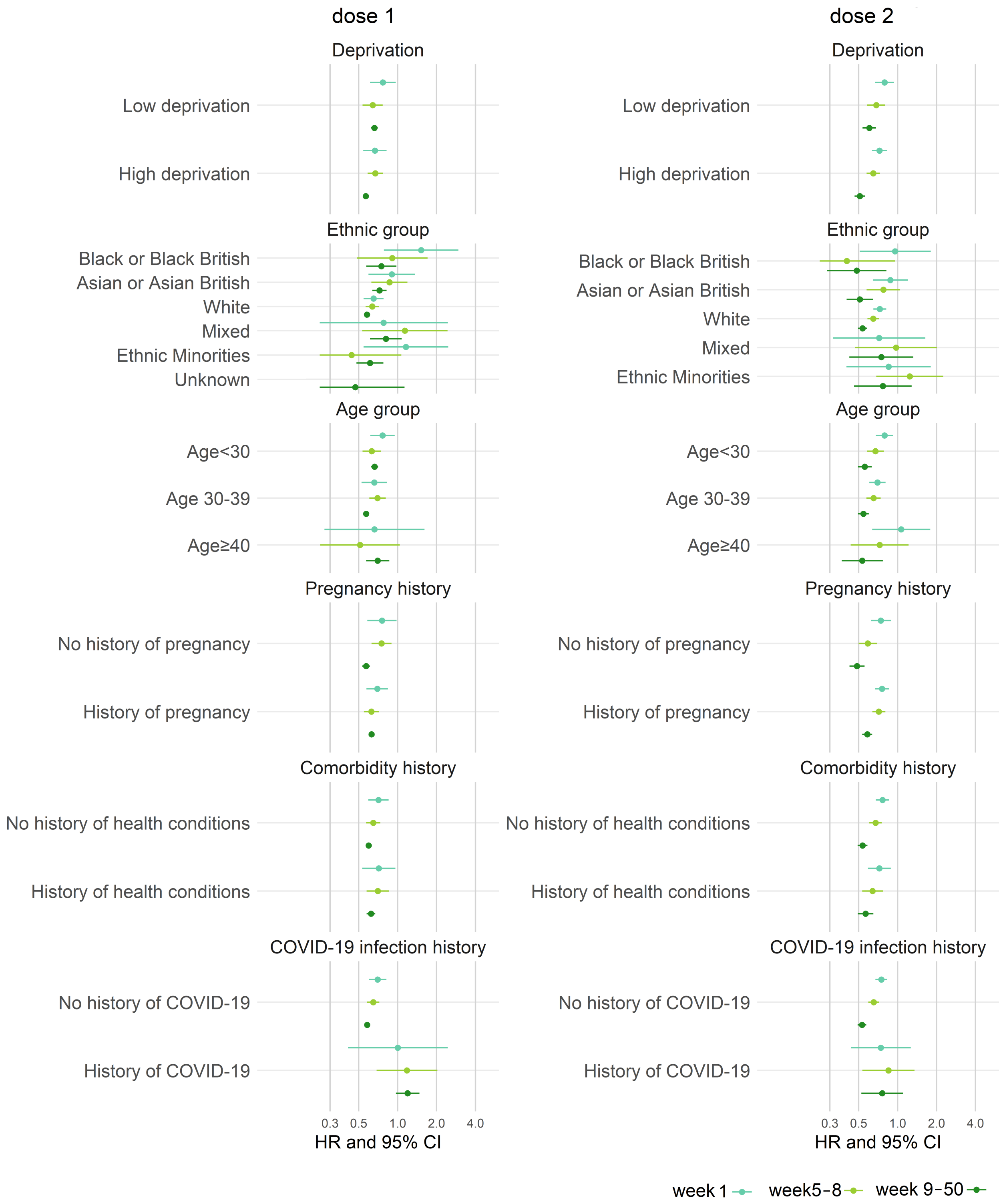

Figure S16. Fully adjusted hazard ratios (log scale) for COVID-19 diagnosis after dose 1 and 2 of COVID-19 vaccine during pregnancy by subgroups (dose 1 analysis n= 148,841, dose 2 analysis n= 57,885, dose 2 analysis includes only England)

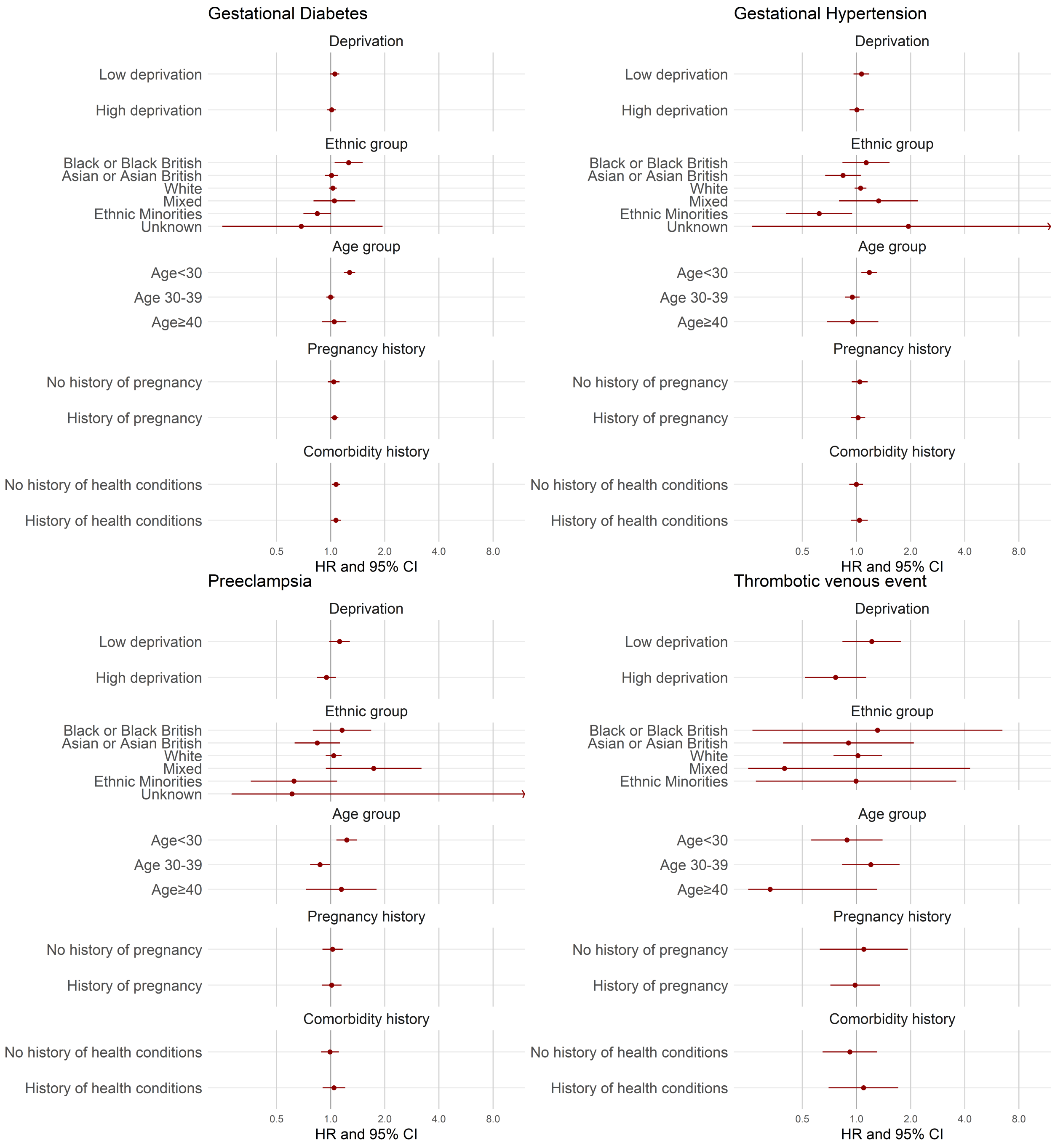

Figure S17. Fully adjusted hazard ratios (log scale) for adverse outcomes during pregnancy after dose 1 of COVID-19 vaccine by subgroups (n=142,035)

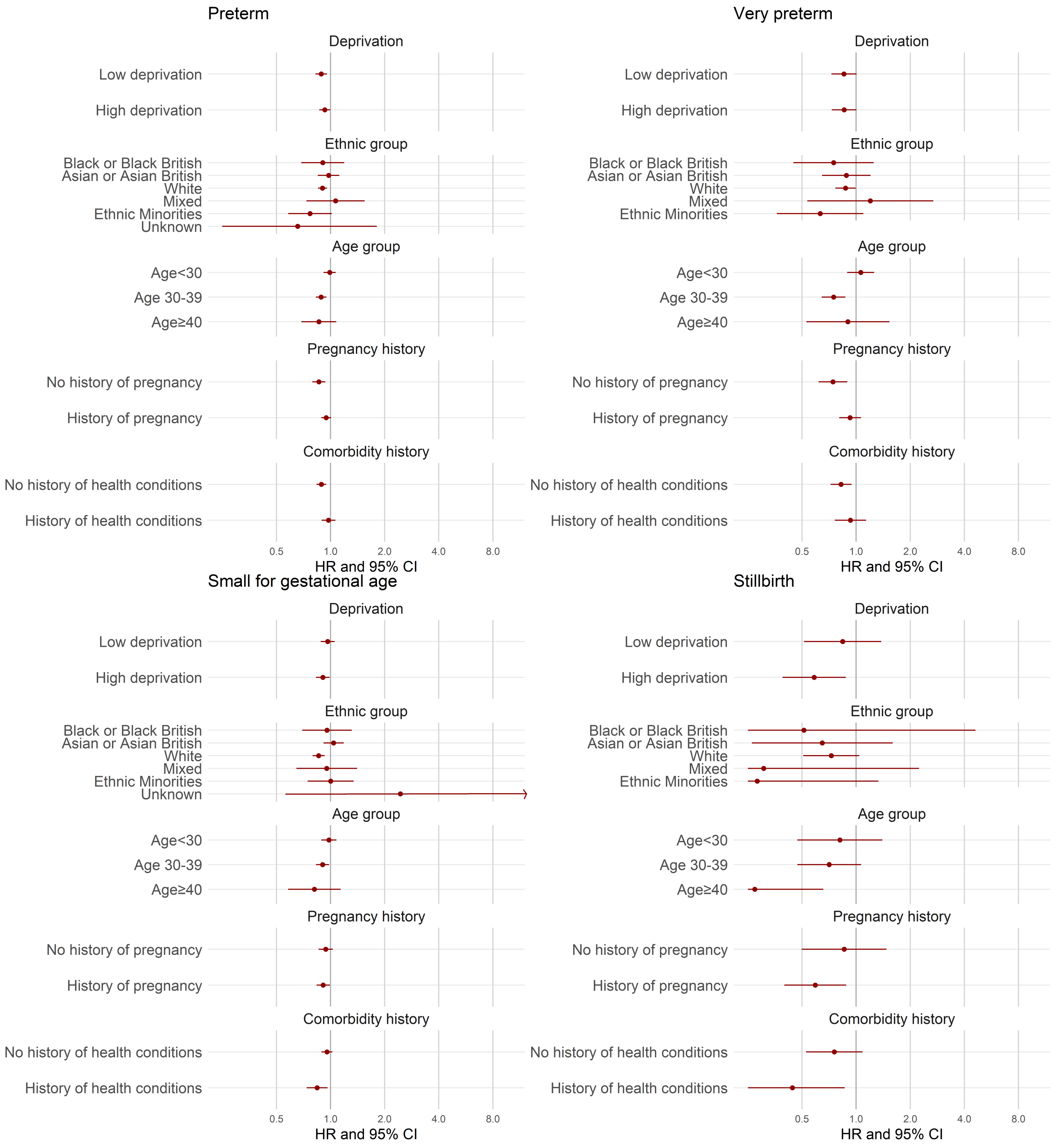

Figure S18. Fully adjusted hazard ratios (log scale) for adverse outcomes at birth after dose 1 of COVID-19 vaccine by subgroups (n=109,845)

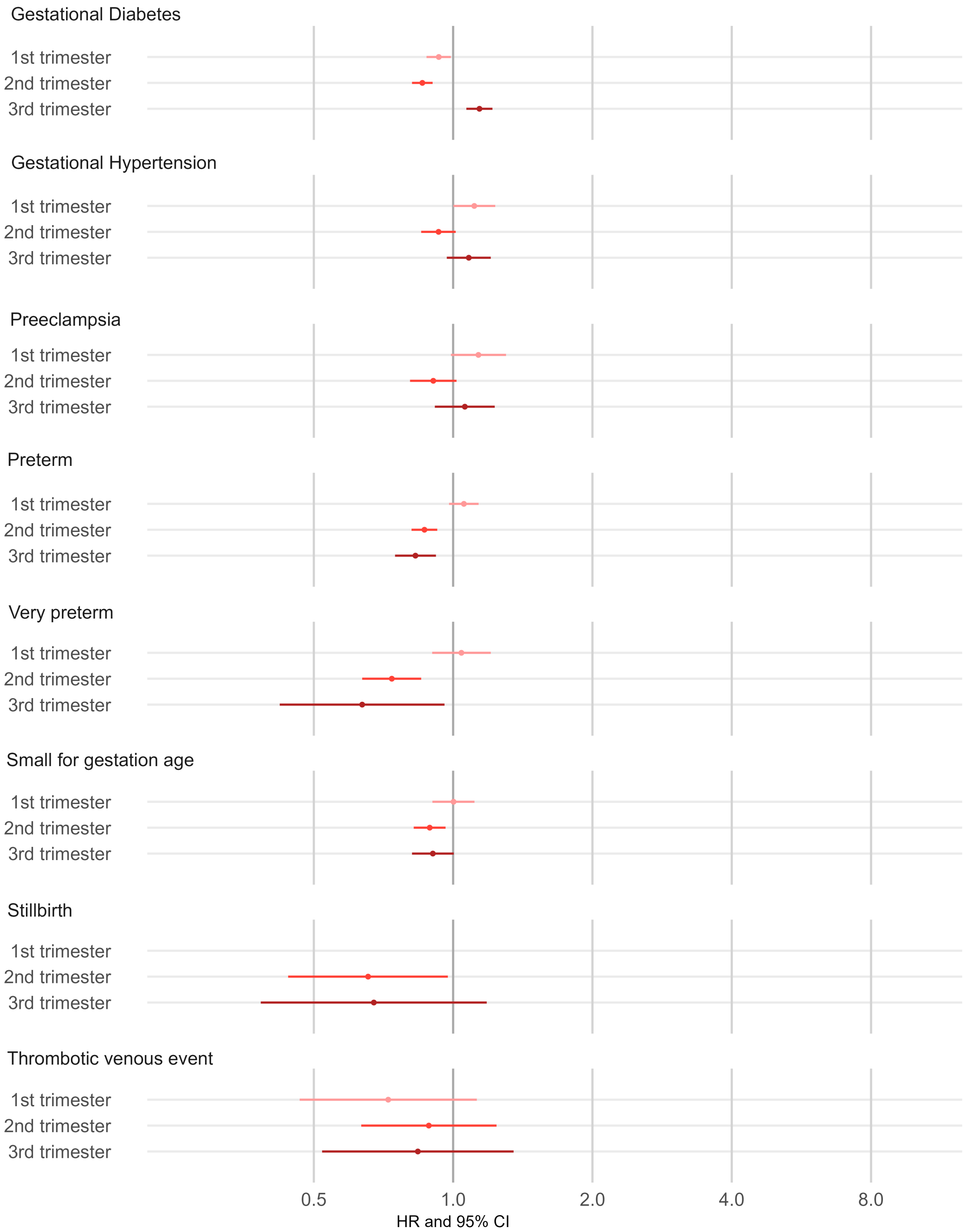

Figure S19. Fully adjusted hazard ratios (log scale) for adverse pregnancy outcomes after dose 1 of COVID-19 vaccine during pregnancy in the overall sample by trimester (n=142,035)

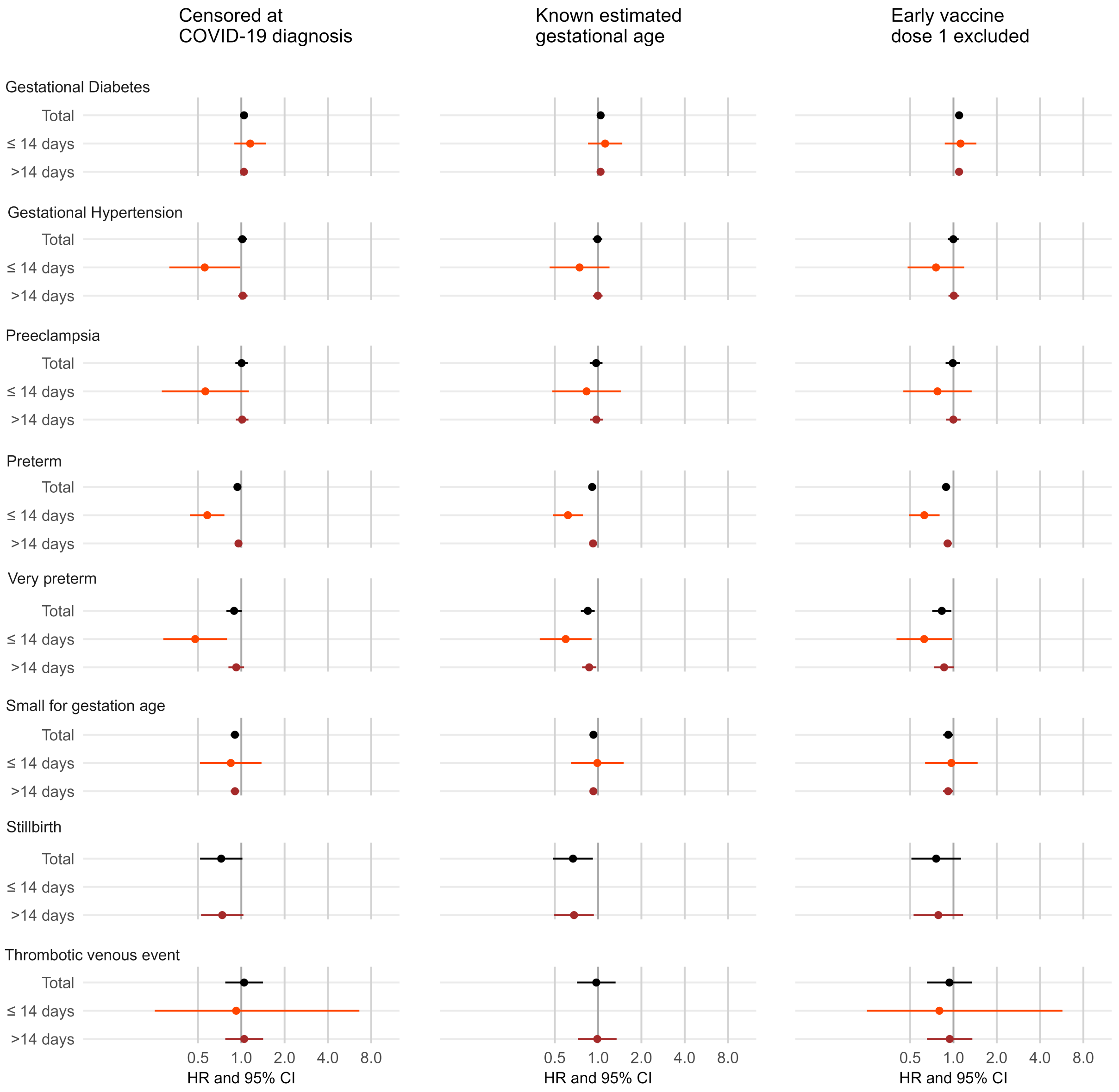

Figure S20. Fully adjusted hazard ratios (log scale) for adverse pregnancy outcomes after dose 1 of COVID-19 vaccine during pregnancy. Sensitivity analyses involved: censoring at COVID-19 diagnosis (n =142,035), restricting the analyses to pregnant women with known estimated gestational age (n=109,845) and excluding early dose 1 of COVID-19 vaccine (before 18th of June 2021) (n=53,060)

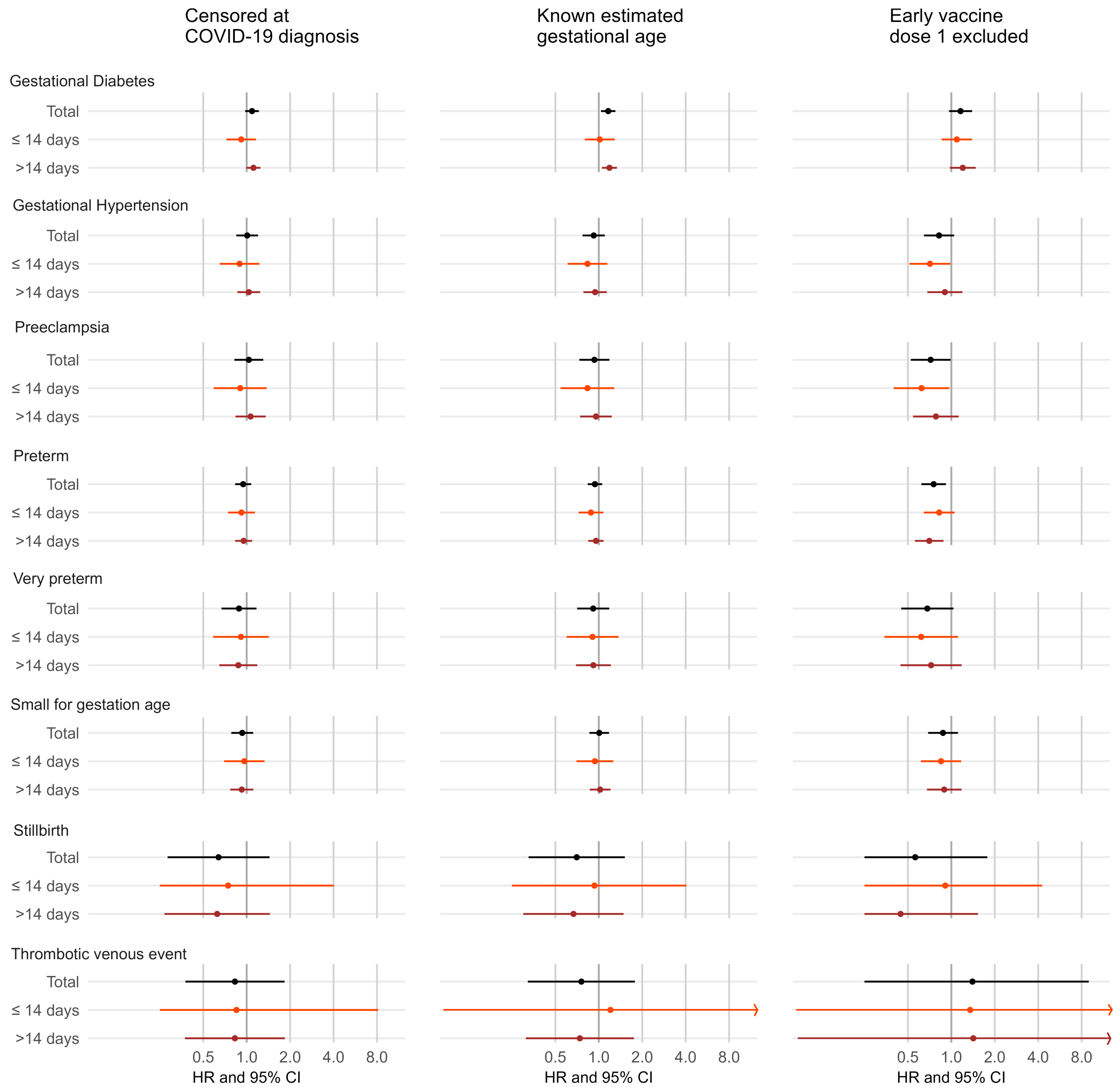

Figure S21. Fully adjusted hazard ratios (log scale) for adverse pregnancy outcomes after dose 2 of COVID-19 vaccine during pregnancy. Sensitivity analyses involved: censoring at COVID-19 diagnosis (n =57,885), restricting the analyses to pregnant women with known estimated gestational age (n=44,600) and excluding early dose 1 of COVID-19 vaccine (before 18th of June 2021) (n=26,935)

Adverse pregnancy outcomes	Description	Sources of data
**Gestational diabetes**	Hyperglycemia (high blood glucose levels) that is first recognized during pregnancy.	Secondary and primary care records (ICD-10 and SNOMED-CT, Read Codes – validated by a clinician)
**Gestational hypertension**	The onset of hypertension (blood pressure ≥ 140/90 mmHg) after 20 weeks of gestation in women who were normotensive before pregnancy.	Secondary and primary care records (ICD-10 and SNOMED-CT, Read Codes – validated by a clinician)
**Preeclampsia**	A multisystem disorder of pregnancy marked by the onset of hypertension and proteinuria or hypertension and significant end-organ dysfunction with or without proteinuria, after the 20th week of gestation.	Secondary and primary care records (ICD-10 and SNOMED-CT, Read Codes – validated by a clinician)
**Stillbirth**	Fetal death that occurs at or beyond 24 weeks of gestation. This includes both deaths occurring before (antepartum death) and during (intrapartum death) delivery.	Secondary and primary care records (ICD-10 and SNOMED-CT, Read Codes – validated by clinician), information on stillbirth at the delivery, for pregnancy with estimated gestational age > 24 weeks
**Preterm birth**	The delivery of an infant before 37 completed weeks of gestation.	< 37 estimated gestational weeks (reported in electronic health records) at delivery
**Very preterm birth**	The delivery of an infant before 32 completed weeks of gestation.	< 32 estimated gestational weeks (reported in electronic health records) at delivery
**Small for gestational age**	A neonate whose birth weight lies below the 5th percentile for their gestational age.	Lower than the 5th percentile of the weight stratified for the gestational week at birth and sex based on a national birth cohort in England of singleton births (1998–2015)
**Venous thrombotic events**	The occurrence of thromboembolic events (formation of a blood clot in a vein) during pregnancy.	Secondary and primary care records (ICD-10 and SNOMED-CT, Read Codes – validated by a clinician)

Table S1. Definition of adverse pregnancy outcomes.

Analysis	Sensitivity analysis	Rationale
**Association between COVID-19 diagnosis and adverse pregnancy outcomes**	Women without recorded estimated gestational age at the delivery	to examine the impact of the magnitude of the association of unrecorded estimated gestational age at delivery (n=200,020, n=199,145 in England and 875 in Wales)
	Women with estimated pregnancy start date < 11th March 2020	to account for unobserved confounding with regards to awareness of the pandemic at conception
	Women with estimated pregnancy start date ≥ 11th March 2020	to account for unobserved confounding with regards to awareness of the pandemic at conception
	Women who had received at least one dose of COVID-19 vaccine up to 31st December 2021	to account for unobserved confounding with regards to vaccination status
	Women who had not received any doses of COVID-19 vaccine up to 31st December 2021	to account for unobserved confounding with regards to vaccination status
	Women who received at least one dose of COVID-19 vaccine from 18th June 2021 to 31st December 2021	to account for unobserved confounding with regards to vaccination status
	Excluding outcomes that occurred starting from day 1 following the exposure	to check whether the cases that occurred on the same day as the exposure have an impact on the effect estimates
	Non-hospitalised COVID-19 diagnosis as the exposure	to check whether effect estimates were driven mainly by severe COVID-19 cases
	Hospitalised COVID-19 diagnosis as the exposure	to estimate the effect estimates in severe COVID-19 cases
	Follow-up censored at the maximal outcome week for preterm and very preterm	to account for bias related to including a COVID-19 diagnosis as an exposure when it occurs after the timeframe in which the outcome could potentially occur (e.g., a COVID-19 diagnosis at 38 weeks of gestation when examining preterm birth as the outcome of interest)
	Women with spontaneous preterm and very preterm	to account for bias, we have excluded non-spontaneous preterm births, as induced labor may be requested due to other conditions that could be influenced by a COVID-19 infection (e.g. preeclampsia)
**Association between COVID-19 vaccination and adverse pregnancy outcomes**	Censoring at COVID-19 diagnosis	to account for confounding with regards to COVID-19 diagnosis
	Women with estimated gestational age at the delivery	to examine the impact of unrecorded estimated gestational age at delivery
	Women vaccinated after 18th June 2021	to account for unobserved confounding with regards to comorbidities and risk of infection, since women vaccinated prior to that date belonged to special high vulnerability groups and/or were frontline healthcare workers

Table S2. Criteria and rationale of sensitivity analyses

	Overall period		
**Characteristics**	**Total n = 865,654 n (%)**	**No COVID-19 infection n = 805,520 n (%)**	**COVID 19 infection n = 60,134 n (%)**
**Age (years)**			
**Median (interquartile range)**	30 (26-34)	30 (26-34)	30 (26-33)
<30	403318 (46.6)	373498 (46.4)	29815 (49.6)
30-39	434849 (50.2)	406301 (50.4)	28548 (47.5)
≥40	27487 (3.2)	25721 (3.2)	1771 (2.9)
**Deprivation**			
1 (least)	214763 (24.8)	198585 (24.7)	16178 (26.9)
2	190090 (22)	176575 (21.9)	13510 (22.5)
3	167387 (19.3)	155955 (19.4)	11432 (19)
4	152663 (17.6)	142275 (17.7)	10388 (17.3)
5 (most)	136868 (15.8)	128487 (16)	8381 (13.9)
Unknown	3883 (0.4)	3648 (0.5)	235 (0.4)
**Ethnic group**			
Black or Black British	40784 (4.7)	38213 (4.7)	2576 (4.3)
Asian or Asian British	105156 (12.1)	96539 (12)	8622 (14.3)
White	647493 (74.8)	603064 (74.9)	44429 (73.9)
Mixed	19219 (2.2)	17921 (2.2)	1298 (2.2)
Ethnic minorities	39968 (4.6)	37565 (4.7)	2403 (4)
Unknown ethnic group	13029 (1.5)	12218 (1.5)	811 (1.3)
**Previous pregnancy**	551787 (63.7)	511781 (63.5)	40006 (66.5)
**COVID-19 diagnosis during pregnancy**	16461 (1.9)	15392 (1.9)	1069 (1.8)
**COVID-19 Hospitalization**	3626 (0.4)		3626 (6)
**Trimester of exposure**			
1st	11250 (1.3)		11250 (18.7)
2nd	18532 (2.1)		18532 (30.8)

3rd	30352 (3.5)		30352 (50.5)
**Dose 1 of COVID-19 vaccine during pregnancy**	98977 (11.4)	91359 (11.3)	7618 (12.7)
**Dose 2 of COVID-19 vaccine during pregnancy**	62478 (7.2)	58293 (7.2)	4180 (7)
**Smoking status**			
Current	152078 (17.6)	142255 (17.7)	9818 (16.3)
Ex	118843 (13.7)	110203 (13.7)	8640 (14.4)
Never	542178 (62.6)	503292 (62.5)	38886 (64.7)
**History of cardiovascular and haematological diseases** [a]	9590 (1.1)	8891 (1.1)	699 (1.2)
**History of hypertension** [a]	84740 (9.8)	77997 (9.7)	6743 (11.2)
**History of diabetes** [a]	44549 (5.1)	41121 (5.1)	3423 (5.7)
**History of depression** [a]	178352 (20.6)	165427 (20.5)	12925 (21.5)
**History of other conditions** [a]	131338 (15.2)	122476 (15.2)	8862 (14.7)

Table S3. Characteristic of the pregnancy cohort: overall period. [a] before pregnancy

	Wales - SAIL			England - SDE		
**Characteristics**	**Total n = 36,474 n (%)**	**No COVID-19 infection n = 34,010 n (%)**	**COVID-19 infection n = 2,464 n (%)**	**Total n = 829,180 n (%)**	**No COVID-19 infection n = 771,510 n (%)**	**COVID-19 infection n = 57,670 n (%)**
**Age**						
**Median (interquartile range)**	29 (25-33)	29 (25-33)	29 (25-33)	30 (26-34)	30 (26-34)	30 (26-33)
<30	19183 (52.6)	17843 (52.5)	1340 (54.4)	384135 (46.3)	355655 (46.1)	28475 (49.4)
30-39	16449 (45.1)	15376 (45.2)	1073 (43.5)	418400 (50.5)	390925 (50.7)	27475 (47.6)
≥40	842 (2.3)	791 (2.3)	51 (2.1)	26645 (3.2)	24930 (3.2)	1720 (3)
**Deprivation**						
1 (least)	8668 (23.8)	8040 (23.6)	628 (25.5)	206095 (24.9)	190545 (24.7)	15550 (27)
2	7205 (19.8)	6700 (19.7)	505 (20.5)	182885 (22.1)	169875 (22)	13005 (22.6)
3	6352 (17.4)	5935 (17.5)	417 (16.9)	161035 (19.4)	150020 (19.4)	11015 (19.1)
4	5628 (15.4)	5250 (15.4)	378 (15.3)	147035 (17.7)	137025 (17.8)	10010 (17.4)
5 (most)	5643 (15.5)	5302 (15.6)	341 (13.8)	131225 (15.8)	123185 (16)	8040 (13.9)
Unknown	2978 (8.2)	2783 (8.2)	195 (7.9)	905 (0.1%)	865 (0.1%)	40 (0.1%)
**Ethnic group**						
Black or Black British	374 (1)	353 (1)	21 (0.9)	40410 (4.9)	37860 (4.9)	2555 (4.4)
Asian or Asian British	981 (2.7)	909 (2.7)	72 (2.9)	104175 (12.6)	95630 (12.4)	8550 (14.8)
White	25973 (71.2)	24224 (71.2)	1749 (71)	621520 (75)	578840 (75)	42680 (74)
Mixed	399 (1.1)	381 (1.1)	18 (0.7)	18820 (2.3)	17540 (2.3)	1280 (2.2)
Ethnic minorities	403 (1.1)	380 (1.1)	23 (0.9)	39565 (4.8)	37185 (4.8)	2380 (4.1)
Unknown ethnic group	8344 (22.9)	7763 (22.8)	581 (23.6)	4685 (0.6)	4455 (0.6)	230 (0.4)
**Previous pregnancy**	28252 (77.5)	26271 (77.2)	1981 (80.4)	523535 (63.1)	485510 (62.9)	38025 (65.9)
**COVID-19 Hospitalization**	111 (0.3)		111 (4.5)	3515 (0.4)		3515 (6.1)
**Trimester of exposure**						
1st	435 (1.2)		435 (17.7)	10815 (1.3)		10815 (18.8)
2nd	722 (2)		722 (29.3)	17810 (2.1)		17810 (30.9)

3rd	1307 (3.6)		1307 (53)	29045 (3.5)		29045 (50.4)
**Dose 1 of COVID-19 vaccine during pregnancy**	4807 (13.2)	4309 (12.7)	498 (20.2)	94170 (11.4)	87050 (11.3)	7120 (12.3)
**Dose 2 of COVID-19 vaccine during pregnancy**	2993 (8.2)	2713 (8)	280 (11.4)	59485 (7.2)	55580 (7.2)	3900 (6.8)
**Smoking status**						
Current	9738 (26.7)	9110 (26.8)	628 (25.5)	142340 (17.2)	133145 (17.3)	9190 (15.9)
Ex	1018 (2.8)	953 (2.8)	65 (2.6)	117825 (14.2)	109250 (14.2)	8575 (14.9)
Never	25718 (70.5)	23947 (70.4)	1771 (71.9)	516460 (62.3)	479345 (62.1)	37115 (64.4)
**History of cardiovascular and haematological diseases** [a]	450 (1.2)	416 (1.2)	34 (1.4)	9140 (1.1)	8475 (1.1)	665 (1.2)
**History of hypertension** [a]	4605 (12.6)	4272 (12.6)	333 (13.5)	80135 (9.7)	73725 (9.6)	6410 (11.1)
**History of diabetes** [a]	1594 (4.4)	1481 (4.4)	113 (4.6)	42955 (5.2)	39640 (5.1)	3310 (5.7)
**History of depression** [a]	10477 (28.7)	9732 (28.6)	745 (30.2)	167875 (20.2)	155695 (20.2)	12180 (21.1)
**History of other conditions** [a]	5258 (14.4)	4946 (14.5)	312 (12.7)	126080 (15.2)	117530 (15.2)	8550 (14.8)

Table S4. Characteristic of the pregnancy cohorts based on NHS SDE and SAIL databank – overall period. [a] before pregnancy

	**Dose 1 of COVID-19 vaccine during pregnancy - analysis**		
**Characteristic**	**Overall, n = 148,841 n (%)**	**No COVID-19 vaccination, n = 87,966 n (%)**	**COVID-19 vaccination, n = 60,875 n (%)**
**Age**			
**Median (interquartile range)**	30 (26-34)	29 (25-33)	32 (29-35)
<30	67986 (45.7)	48240 (54.8)	19746 (32.4)
30-39	75871 (51)	37362 (42.5)	38509 (63.3)
≥40	4979 (3.3)	2364 (2.7)	2615 (4.3)
**Deprivation**			
1 (least)	36257 (24.4)	26833 (30.5)	9419 (15.5)
2	32656 (21.9)	21109 (24)	11547 (19)
3	28603 (19.2)	16140 (18.3)	12463 (20.5)
4	26601 (17.9)	13330 (15.2)	13271 (21.8)
5 (most)	24003 (16.1)	10161 (11.6)	13842 (22.7)
Unknown	716 (0,5)	383 (0,4)	328 (0,5)
**Ethnic group**			
Black or Black British	7405 (5)	5950 (6.8)	1455 (2.4)
Asian or Asian British	18285 (12.3)	11771 (13.4)	6514 (10.7)
White	110931 (74.5)	62333 (70.9)	48598 (79.8)
Mixed	3522 (2.4)	2384 (2.71)	1138 (1.87)
Ethnic minorities	6582 (4.4)	4222 (4.8)	2360 (3.9)
Unknown ethnic group	2111 (1.4)	1306 (1.5)	805 (1.3)
**Previous pregnancy**	93422 (62.8)	56009 (63.7)	37413 (61.5)
**Smoking status**			
Current	25408 (17.1)	18731 (21.3)	6677 (11)
Ex	20019 (13.4)	11359 (12.9)	8660 (14.2)
Never	93159 (62.6)	50061 (56.9)	43098 (70.8)

**History of cardiovascular and hematological diseases** [a]	1542 (1)	885 (1)	657 (1.1)
**History of hypertension** [a]	15508 (10.4)	9178 (10.4)	6330 (10.4)
**History of diabetes** [a]	7278 (4.9)	3956 (4.5)	3322 (5.5)
**History of depression** [a]	30664 (20.6)	18553 (21.1)	12111 (19.9)
**History of other conditions** [a]	16120 (10.8)	9368 (10.6)	6752 (11.1)

Table S5. Characteristics of the pregnancy cohort in the vaccination era without dose 1 of COVID-19 vaccine before pregnancy in England and Wales. [a] year before pregnancy

	Dose 1 of COVID-19 vaccine during pregnancy			Dose 2 of COVID-19 vaccine during pregnancy		
**Adverse pregnancy outcomes**	**Follow-up (person-years)**	**N events**	**IR**	**Follow-up (person-years)**	**N events**	**IR**
**Gestational diabetes**	109398.7	11920	108.96 (107.00, 110.92)	42543.8	5030	118.23 (114.96, 121.50)
**Gestational hypertension**	111230.5	4355	39.15 (37.99, 40.32)	43373.4	1795	41.38 (39.47, 43.32)
**Pre-eclampsia**	111317.1	2470	22.19 (21.32, 23.07)	43407.2	990	22.81 (21.40, 24.24)
**Preterm birth** [a]	86543.5	8605	99.43 (97.34, 101.53)	33236.2	2840	85.45 (82.32, 88.61)
**Very preterm birth**[a]	86543.5	1860	21.49 (20.52, 22.47)	33236.2	515	15.5 (14.17, 16.85)
**Small for gestational age** [a]	86543.5	5830	67.36 (65.64, 69.10)	33236.2	1905	57.32 (54.76, 59.90)
**Stillbirth** [a]	81600.5	215	2.63 (2.29, 2.99)	33236.2	70	2.11 (1.62, 2.62)
**Thrombotic venous event**	106210.8	250	2.35 (2.06, 2.65)	43388.3	100	2.3 (1.87, 2.77)

Table S6. Incidence of adverse pregnancy outcomes by pandemic period, follow-up, total number of events incidence rate (IR) per 1000 person-years, (dose 1 analysis n= 148,841, dose 2 analysis n= 57,885, dose 2 analysis includes only England). [a] excluding women without a recorded estimated gestational age at delivery

	Dose 2 of COVID-19 vaccine during pregnancy - analysis		
**Characteristic**	**Overall, n = 57,885 n (%)**	**No COVID-19 vaccination, n = 12,650 n (%)**	**COVID-19 vaccination, n = 45,235 n (%)**
**Age**			
**Median (interquartile range)**	32 (28-35)	30 (26-34)	32 (29-35)
<30	18465 (32)	5840 (46)	12630 (28)
30-39	36885 (64)	6385 (50)	30505 (67)
≥40	2530 (4.4)	425 (3.4)	2105 (4.7)
**Deprivation**			
1 (least)	8880 (15)	2935 (23)	5950 (13)
2	10990 (19)	2730 (22)	8260 (18)
3	11900 (21)	2515 (20)	9385 (21)
4	12740 (22)	2405 (19)	10335 (23)
5 (most)	13320 (23)	2050 (16)	11270 (25)
Unknown	716 (0,5)	383 (0,4)	328 (0,5)
**Ethnic group**			
Black or Black British	1430 (2.5)	510 (4.0)	920 (2.0)
Asian or Asian British	6430 (11)	1770 (14)	4660 (10)
White	46400 (80)	9655 (76)	36745 (81)
Mixed	1110 (1.9)	220 (1.7)	890 (2.0)
Ethnic minorities	2330 (4.0)	455 (3.6)	1875 (4.1)
Unknown ethnic group	180 (0.3)	40 (0.3)	140 (0.3)
**Previous pregnancy**	35150 (61)	8095 (64)	27050 (60)
**Smoking status**			
Current	6110 (11)	2095 (18)	4015 (9.2)
Ex	8595 (16)	1875 (16)	6720 (15)
Never	40740 (73)	7920 (67)	32820 (75)

**History of cardiovascular and hematological diseases** [a]	630 (1.1)	170 (1.4)	460 (1.0)
**History of hypertension** [a]	5965 (10)	1505 (12)	4460 (9.9)
**History of diabetes** [a]	3195 (5.5)	850 (6.7)	2345 (5.2)
**History of depression** [a]	11345 (20)	2995 (24)	8350 (18)
**History of other conditions** [a]	6465 (11)	1545 (12)	4915 (11)

Table S7. Characteristic of the pregnancy cohort: dose 2 analysis (only England). [a] before pregnancy

	Item No.	STROBE items	Location in manuscript	RECORD items	Location in manuscript
**Title and abstract**					
	1	(a) Indicate the study's design with a commonly used term in the title or the abstract (b) Provide in the abstract an informative and balanced summary of what was done and what was found	Page 1	RECORD 1.1: The type of data used should be specified in the title or abstract. When possible, the name of the databases used should be included.<br><br>RECORD 1.2: If applicable, the geographic region and timeframe within which the study took place should be reported in the title or abstract.<br><br>RECORD 1.3: If linkage between databases was conducted for the study, this should be clearly stated in the title or abstract.	Page 1
**Introduction**					
Background rationale	2	Explain the scientific background and rationale for the investigation being reported	Pages 2-3		
Objectives	3	State specific objectives, including any prespecified hypotheses	Page 3		
**Methods**					
Study Design	4	Present key elements of study design early in the paper	Page 3		
Setting	5	Describe the setting, locations, and relevant dates, including periods of recruitment, exposure, follow-up, and data collection	Pages 3-4<br>Table S1		
Participants	6	*(a) Cohort study* - Give the eligibility criteria, and the sources and methods of selection of participants. Describe methods of follow-up<br>*Case-control study* - Give the eligibility criteria, and the sources and methods of	Pages 3-5<br>Supplementary methods	RECORD 6.1: The methods of study population selection (such as codes or algorithms used to identify subjects) should be listed in detail. If this is not possible, an explanation should be provided.	Pages 3-5<br><br>Table S1<br><br>Supplementary methods

		case ascertainment and control selection. Give the rationale for the choice of cases and controls<br>*Cross-sectional study* - Give the eligibility criteria, and the sources and methods of selection of participants<br><br>*(b) Cohort study* - For matched studies, give matching criteria and number of exposed and unexposed<br>*Case-control study* - For matched studies, give matching criteria and the number of controls per case		RECORD 6.2: Any validation studies of the codes or algorithms used to select the population should be referenced. If validation was conducted for this study and not published elsewhere, detailed methods and results should be provided.<br><br>RECORD 6.3: If the study involved linkage of databases, consider use of a flow diagram or other graphical display to demonstrate the data linkage process, including the number of individuals with linked data at each stage.	Github repository<br><br>Figure S1
Variables	7	Clearly define all outcomes, exposures, predictors, potential confounders, and effect modifiers. Give diagnostic criteria, if applicable.	Pages 3-5<br>Supplementary methods<br>Table S1	RECORD 7.1: A complete list of codes and algorithms used to classify exposures, outcomes, confounders, and effect modifiers should be provided. If these cannot be reported, an explanation should be provided.	Pages 3-5<br>Supplementary methods<br>Table S1<br>Github repository
Data sources/ measurement	8	For each variable of interest, give sources of data and details of methods of assessment (measurement).<br>Describe comparability of assessment methods if there is more than one group	Pages 3-5		
Bias	9	Describe any efforts to address potential sources of bias	Pages 4-5<br>Table S2		
Study size	10	Explain how the study size was arrived at	Figure S1		
Quantitative variables	11	Explain how quantitative variables were handled in the analyses. If applicable, describe which groupings were chosen, and why	Pages 4-5		
Statistical methods	12	(a) Describe all statistical methods, including those used to control for confounding	Pages 4-5<br>Table S3		

		(b) Describe any methods used to examine subgroups and interactions<br>(c) Explain how missing data were addressed<br>(d) *Cohort study* - If applicable, explain how loss to follow-up was addressed<br>*Case-control study* - If applicable, explain how matching of cases and controls was addressed<br>*Cross-sectional study* - If applicable, describe analytical methods taking account of sampling strategy<br>(e) Describe any sensitivity analyses			
Data access and cleaning methods		..		RECORD 12.1: Authors should describe the extent to which the investigators had access to the database population used to create the study population.<br><br>RECORD 12.2: Authors should provide information on the data cleaning methods used in the study.	Pages 5 and 11
Linkage		..		RECORD 12.3: State whether the study included person-level, institutional-level, or other data linkage across two or more databases. The methods of linkage and methods of linkage quality evaluation should be provided.	Page 3 Supplementary methods
**Results**					
Participants	13	(a) Report the numbers of individuals at each stage of the study (*e.g.*, numbers potentially eligible, examined for eligibility, confirmed eligible, included in the study, completing follow-up, and analysed)<br>(b) Give reasons for non-participation at each stage.<br>(c) Consider use of a flow diagram	Figure S1<br>Pages 5-6	RECORD 13.1: Describe in detail the selection of the persons included in the study (*i.e.,* study population selection) including filtering based on data quality, data availability and linkage. The selection of included persons can be described in the text and/or by means of the study flow diagram.	Figure S1<br>Pages 5-6
Descriptive data	14	(a) Give characteristics of study participants (*e.g.*, demographic, clinical,	Pages 5-7<br>Table 1, S3, S4, S5 and S7		

		social) and information on exposures and potential confounders<br>(b) Indicate the number of participants with missing data for each variable of interest<br>(c) *Cohort study* - summarise follow-up time (*e.g.*, average and total amount)			
Outcome data	15	*Cohort study* - Report numbers of outcome events or summary measures over time<br>*Case-control study* - Report numbers in each exposure category, or summary measures of exposure<br>*Cross-sectional study* - Report numbers of outcome events or summary measures	Table 2 and Table S6<br>Pages 5-7		
Main results	16	(a) Give unadjusted estimates and, if applicable, confounder-adjusted estimates and their precision (e.g., 95% confidence interval). Make clear which confounders were adjusted for and why they were included<br>(b) Report category boundaries when continuous variables were categorized<br>(c) If relevant, consider translating estimates of relative risk into absolute risk for a meaningful time period	Figure 1, Figure 2 and 3<br>Pages 5-8		
Other analyses	17	Report other analyses done—e.g., analyses of subgroups and interactions, and sensitivity analyses	Figure S2-S20		
**Discussion**					
Key results	18	Summarise key results with reference to study objectives	Page 9		
Limitations	19	Discuss limitations of the study, taking into account sources of potential bias or imprecision. Discuss both direction and magnitude of any potential bias	Page 10	RECORD 19.1: Discuss the implications of using data that were not created or collected to answer the specific research question(s). Include discussion of misclassification bias, unmeasured confounding, missing data, and changing	Page 10

				eligibility over time, as they pertain to the study being reported.	
Interpretation	20	Give a cautious overall interpretation of results considering objectives, limitations, multiplicity of analyses, results from similar studies, and other relevant evidence	Page 10		
Generalisability	21	Discuss the generalisability (external validity) of the study results	Page 10		
**Other Information**					
Funding	22	Give the source of funding and the role of the funders for the present study and, if applicable, for the original study on which the present article is based	Pages 5 and 11		
Accessibility of protocol, raw data, and programming code		..		RECORD 22.1: Authors should provide information on how to access any supplemental information such as the study protocol, raw data, or programming code.	Page 11 Github repository

The RECORD statement – checklist of items, extended from the STROBE statement, that should be reported in observational studies using routinely collected health data.
